# Solid-State ^19^F NMR Chemical Shift in Square-Planar Nickel–Fluoride Complexes Linked by Halogen Bonds

**DOI:** 10.1021/acs.inorgchem.2c04063

**Published:** 2023-03-15

**Authors:** Abril C. Castro, Michele Cascella, Robin N. Perutz, Christophe Raynaud, Odile Eisenstein

**Affiliations:** † Hylleraas Centre for Quantum Molecular Sciences, Department of Chemistry, University of Oslo, 0315 Oslo, Norway; ‡ Department of Chemistry, University of York, Heslington, YO10 5DD York, United Kingdom; § ICGM, Université Montpellier, CNRS, ENSCM, 34090 Montpellier, France

## Abstract

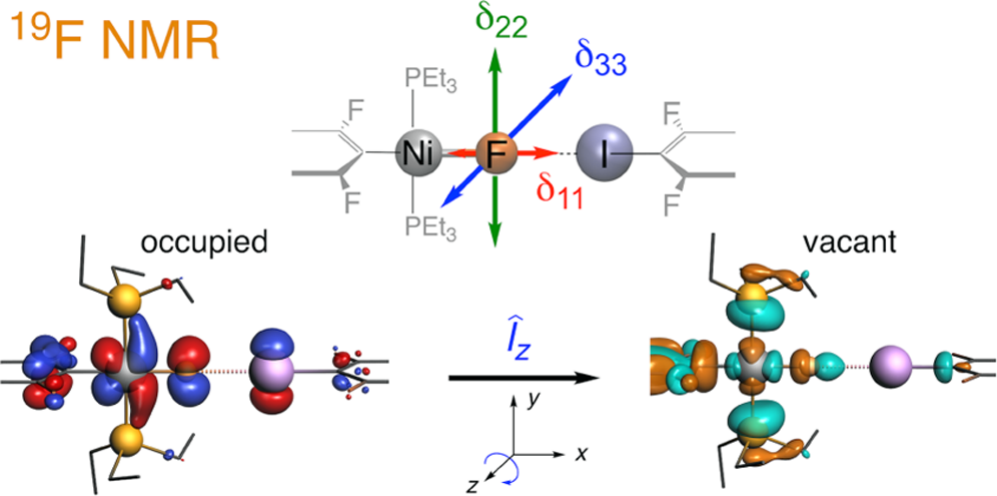

The halogen bond (XB) is a highly directional class of noncovalent interactions widely explored by experimental and computational studies. However, the NMR signature of the XB has attracted limited attention. The prediction and analysis of the solid-state NMR (SSNMR) chemical shift tensor provide useful strategies to better understand XB interactions. In this work, we employ a computational protocol for modeling and analyzing the ^19^F SSNMR chemical shifts previously measured in a family of square-planar *trans* Ni^II^-L_2_-iodoaryl-fluoride (L = PEt_3_) complexes capable of forming self-complementary networks held by a NiF···I(C) halogen bond [ThangavadivaleV.; Chem. Sci.2018, 9, 3767−37812978050910.1039/c8sc00890fPMC5939608]. To understand how the ^19^F NMR resonances of the nickel-bonded fluoride are affected by the XB, we investigate the origin of the shielding in *trans*-[NiF(2,3,5,6-C_6_F_4_I)(PEt_3_)_2_], *trans*-[NiF(2,3,4,5-C_6_F_4_I)(PEt_3_)_2_], and *trans*-[NiF(C_6_F_5_)(PEt_3_)_2_] in the solid state, where a XB is present in the two former systems but not in the last. We perform the ^19^F NMR chemical shift calculations both in periodic and molecular models. The results show that the crystal packing has little influence on the NMR signatures of the XB, and the NMR can be modeled successfully with a pair of molecules interacting via the XB. Thus, the observed difference in chemical shift between solid-state and solution NMR can be essentially attributed to the XB interaction. The very high shielding of the fluoride and its driving contributor, the most shielded component of the chemical shift tensor, are well reproduced at the 2c-ZORA level. Analysis of the factors controlling the shielding shows how the highest occupied Ni/F orbitals shield the fluoride in the directions perpendicular to the Ni–F bond and specifically perpendicular to the coordination plane. This shielding arises from the magnetic coupling of the Ni(3d)/F(2p lone pair) orbitals with the vacant σ_Ni–F_^*^ orbital, thereby rationalizing the very highly upfield (shielded) resonance of the component (δ_33_) along this direction. We show that these features are characteristic of square-planar nickel–fluoride complexes. The deshielding of the fluoride in the halogen-bonded systems is attributed to an increase in the energy gap between the occupied and vacant orbitals that are mostly responsible for the paramagnetic terms, notably along the most shielded direction.

## Abstract 2

The calculated ^19^F NMR resonances in nickel−fluoride complexes involved in halogen bonding reproduce values observed by SSNMR. Analysis shows how the occupied Ni/F orbitals shield F in directions perpendicular to Ni−F. Shielding arises from the magnetic coupling of the occupied orbitals with vacant σ_Ni−F_^*^, thereby rationalizing the very highly shielded resonance of δ_33_. Deshielding of F in the halogen-bonded systems is attributed to an increase in the energy gap between the involved orbitals.

## Introduction

The halogen bond (XB) is a stabilizing noncovalent interaction involving halogen atoms. It is constituted by an electrophilic region, associated with a covalently bonded halogen atom called the XB donor, and a nucleophilic region, typically a Lewis base, called the XB acceptor.^1^ Similar in energy to the more prominent hydrogen bonds,^2,3^ XBs form highly directional interactions, exploited by many applications in supramolecular chemistry, crystal engineering, materials design, and biological systems.^4−6^ The nature of the XB, which has attracted considerable interest from the theoretical community, has been debated for many years.^7−17^ Clark, et al. introduced the concept of the σ-hole, that is, the emergence of a region of positive electrostatic potential along the extension of the covalently bonded halogen atom, to account for the electrophilic behavior of a halogen in an attractive interaction toward diverse Lewis bases.^18^ However, this concept could not describe XBs in full. Numerous theoretical studies have shown that charge transfer, electrostatics, dispersion, and polarization interactions contribute to the XB.^8,9,14−17,19−21^

While the XB has been probed by diverse spectroscopic techniques,^22−26^ nuclear magnetic resonance (NMR) spectroscopy has been especially useful as it can detect the interaction either in solution^6^ or in the solid state,^5^ both for organic molecules^27,28^ and for transition-metal-containing systems.^29^ Due to the high XB donor strength of iodine, the majority of the studies have been carried out with an organic species bearing a C–I bond, while a wide variety of Lewis bases have been used as XB acceptor. Overall, the ^13^C NMR resonance of the C–I bond is deshielded by the XB, while the chemical shifts of the XB acceptors depend on their molecular nature. However, using the C–I bond length as criterion, a correlation between chemical shifts and XB strength was established.^5^ In contrast, no clear relationship appeared between NMR chemical shifts and distances between the XB donor and acceptor, even though the chemical shifts of the atoms involved in the XB are known to be sensitive to structural features.^30−32^ So far, few studies concerned the relationship between the NMR chemical shift at atoms involved in the XB and variations in molecular properties of the XB donor and acceptor.^33^

**Figure 1:**
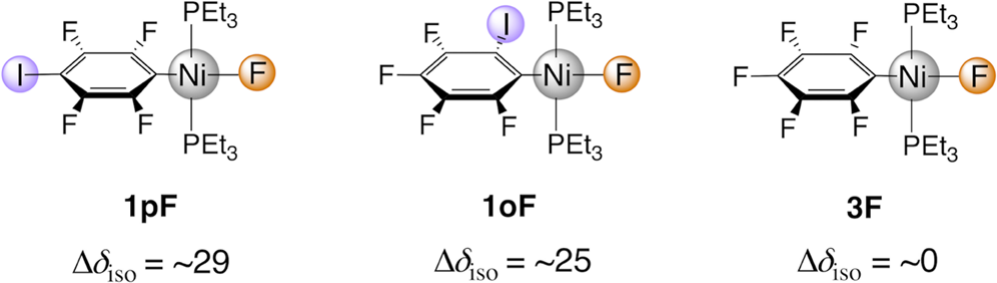
Nickel–fluoride complexes considered in this work.^39,42^ The experimental Δδ_iso_ values (in ppm) give the difference between the solid-state and solution ^19^F NMR resonances of the nickel-bonded fluoride. The labels used in ref (39) are kept for easier connection with this work.

The fact that metal–fluoride complexes can form strong hydrogen bonds and XBs demonstrated that the fluoride can act as a potent Lewis base and that the thermodynamics of the XB was measurable and highly sensitive to the chemical nature of the metal–ligand complex.^34−38^ Investigations of XB interactions involving metal centers are thus particularly useful because their strength can be tuned by changing either the inorganic halogen (M–X) acting as XB acceptor or the organic halogen (C–X′) acting as XB donor.^29^ Furthermore, the metal–fluoride complexes are of special interest because they exhibit a ^19^F NMR resonance that lies upfield away from all of the other ^19^F resonances and is acutely responsive to the chemical environment. Importantly, the fluorine resonance is deshielded in the presence of a XB by up to 25–40 ppm and can thus be an ideal reporter for the XB characteristics. In particular, the solid-state NMR (SSNMR) study of Ni^II^–fluoride complexes that form self-complementary networks^39^ offers information on the anisotropy of the fluorine chemical shift; comparable ^19^F SSNMR measurements are rather rare.^40,41^ The effect of the XB on the ^19^F SSNMR chemical shift was demonstrated by comparing the NMR spectra of *trans*-[NiF(2,3,5,6-C_6_F_4_I)(PEt_3_)_2_] **1pF** and *trans*-[NiF(2,3,4,5-C_6_F_4_I)(PEt_3_)_2_] **1oF** complexes, with that of a complex incapable of XB formation, *trans*-[NiF(C_6_F_5_)(PEt_3_)_2_] **3F**(42) (Figure 1). For **1pF** and **1oF**, the presence of the XB is manifested through deshielding of the nickel-bonded ^19^F SSNMR isotropic chemical shifts (δ_iso_), as has been reported in solution with organic XB donors.^34−38^ Comparisons between solid-state and solution NMR indicated that NiF···I(C) halogen bond leads to a deshielding of 25–29 ppm (Δδ_iso_, Figure 1). This deshielding is caused essentially by the XB effect since **3F** showed almost the same chemical shift in the solid state and in solution.

The NMR chemical shift anisotropy, represented by the chemical shift tensor (CST), can be used to understand the effect of the XB on δ_iso_. The principal components of the CST (δ_11_, δ_22_, δ_33_ with δ_11_ ≥ δ_22_ ≥ δ_33_) have been shown to be controlled mainly by the paramagnetic terms, which themselves can be understood on the basis of the couplings between occupied and vacant molecular orbitals (MOs) having contributions on the NMR active atom and its immediate environment. This analysis has provided valuable insight into the electronic structure of various molecules and their reactivity,^43−54^ but studies of halogen-bonded systems are rare. A pioneer example is the analysis of the ^13^C NMR shielding tensor of the C–I bond in diiodoacetylene acting as XB donor with amines,^55^ where the observed and calculated deshielding at the iodine-bonded carbon was traced back to a contribution from the paramagnetic coupling of the occupied π MOs of the alkyne with the vacant σ_C–I_^*^ MO. Later, a theoretical analysis of halide quadrupolar coupling tensors also pointed to the key role of σ_C–I_^*^ on the XB acceptor.^30^ The chemical shift of ^15^N NMR of pyridine acting as a XB acceptor was also interpreted qualitatively by the variation in the energy of the occupied and vacant MOs of pyridine under the influence of the XB.^56^

Preliminary nonrelativistic (NR) calculations using a model complex of **1pF** halogen-bonded to IC_6_F_5_ gave ^19^F NMR resonances in reasonable agreement with the experimental data.^39^ The main effect of the XB is the deshielding of δ_33_, which in turn is the most shielded component of the CST. The variation in δ_33_ component follows the F···I distance qualitatively and mirrors the variation in δ_iso_. Nevertheless, a detailed analysis of the CST was not carried out; neither is the ranking of the principal components of the CST understood nor is the effect of the XB on them. In this work, we will fill this gap in knowledge.

## Results and Discussion

### Structural Features

**Figure 2:**
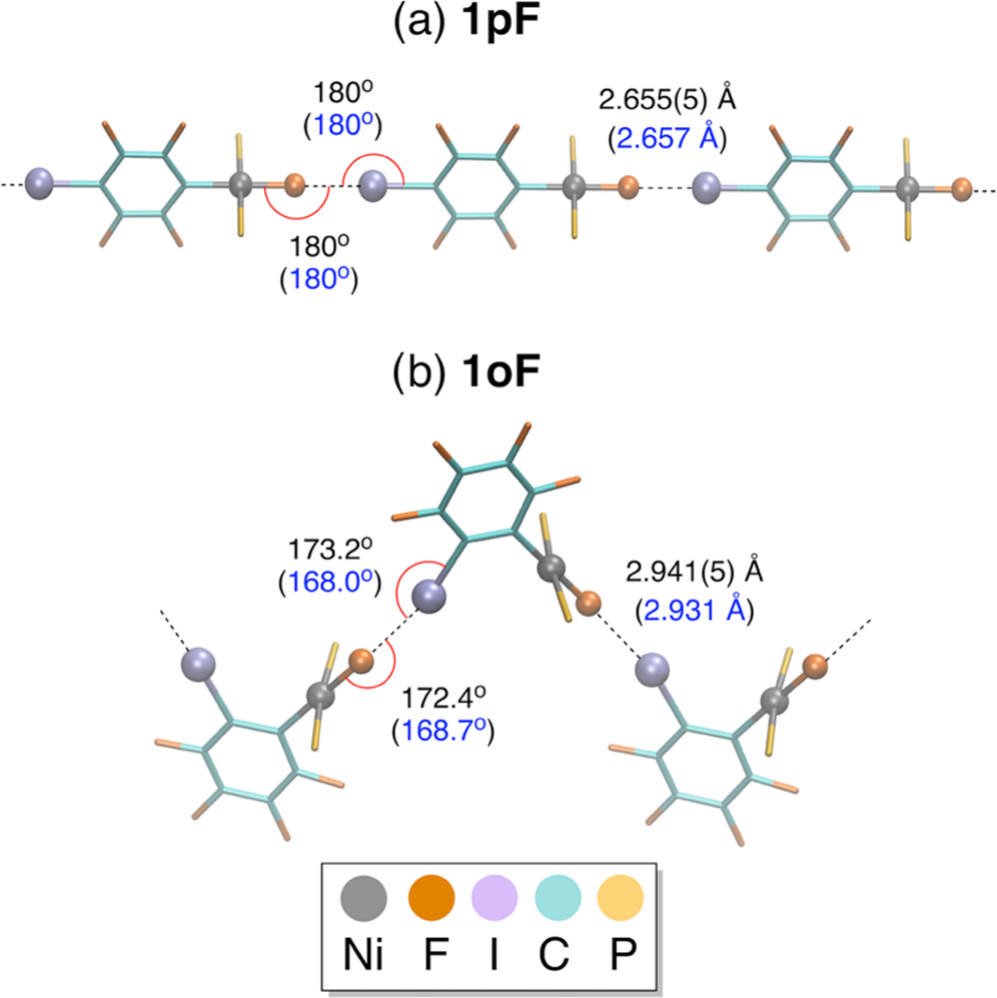
Selected XB structural features for (a) **1pF** and (b) **1oF** (ethyl groups of the PEt_3_ ligands perpendicular to the aryl plane omitted), from X-ray diffraction analysis in black,^39^ and from calculations with periodic lattice models in blue (this work).

The reported crystal structures of **1pF** and **1oF** reveal, respectively, linear or zigzag chains, linked by XBs (Figure 2 and Tables S1–S3 for a representation of the unit cells). The XB environment is similar in the two complexes containing a nickel fluoride as the XB acceptor and a coordinated C_6_F_4_I ligand as the XB donor. The C–I···F and Ni–F···I angles of **1pF** are perfectly linear (Figure 2a), the first one being consistent with typical XB behavior. This preference for a linear arrangement is also observed for **1oF**, as shown by the C–I···F and Ni–F···I angles of 173.2(2) and 172.4(3)°, respectively (Figure 2b). Note that the NiF···I(C) XB distance is significantly shorter for **1pF**, 2.655(5) Å, than for **1oF**, 2.941(5) Å. On the other hand, **3F** itself, which has no iodine on the aryl ligand, does not form a XB. The Ni–F bond distance of 1.838(6) Å in **3F** resembles that in **1pF** (1.837(5) Å) and **1oF** (1.841(5) Å). Likewise, the plane of the aryl C_6_F_4_I or C_6_F_5_ ligand is almost perpendicular to the Ni plane in the three complexes, as shown by the dihedral angles of 85.2(2)° in **1pF**, 84.4(2)° in **1oF**, and 89.2(2)° in **3F**.^39,42^

**Scheme 1:**
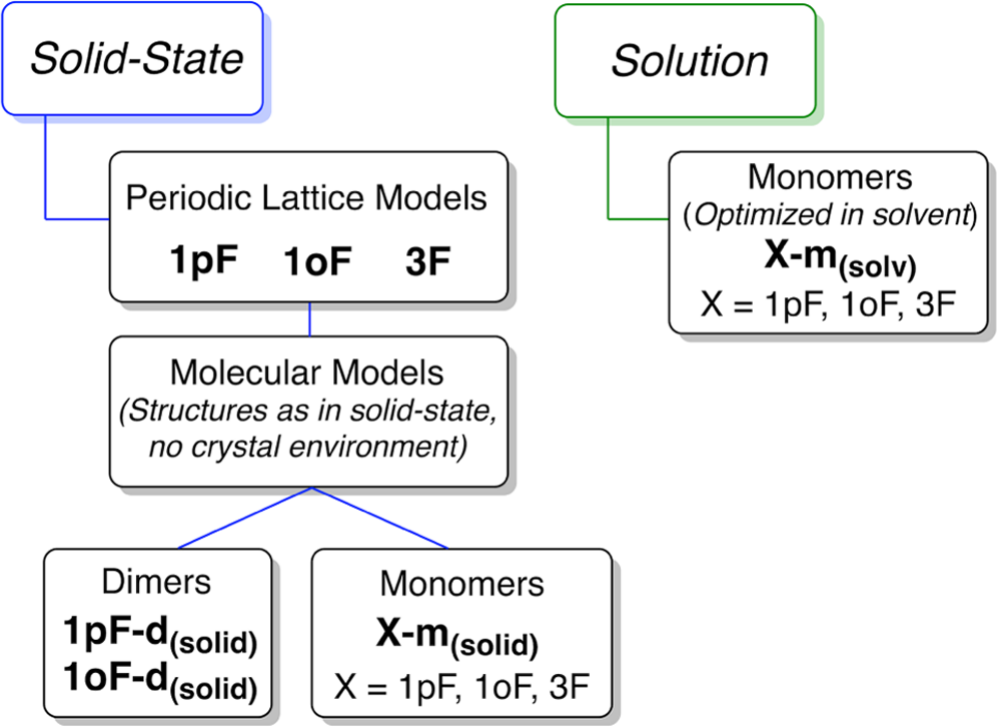
Nomenclature Used in This Study The experimental systems are also labeled **1pF**, **1oF**, and **3F**, as in the original article.^39^

Several models were used in this study and their associated labeling is given in Scheme 1. Periodic lattice models were used to include a full representation of the crystalline solid in **1pF**, **1oF**, and **3F**. The benchmark study shows the computed (*a*, *b*, *c*) unit-cell parameters in reasonable agreement with the experimental data, with root-mean-square deviations (RMSDs) of up to 0.43 Å (Tables S1–S3). All functionals considered exhibit similar results, although the unit-cell parameters are slightly better reproduced including the Grimme’s dispersion D3 correction^57^ (see the Supporting Information, SI). However, the PBE0/pob_TZVP^58^ level provides the best reproduction of the structural parameters for the three nickel complexes (Tables S4–S6). In particular, this level of theory accurately reproduces the NiF···I(C) XB distances; the calculated NiF···I(C) XB lengths of 2.657 and 2.931 Å for **1pF** and **1oF**, respectively, are only 0.002 and 0.010 Å longer than in the experiment, within the limits of 3 standard deviations (conventionally taken as significance limits) (Figure 2). Hence, the significantly shorter XB interaction for **1pF** compared to **1oF** is correctly reproduced. The calculated Ni–F bond distances of **1pF** and **3F** are also well within 3 standard deviations of the experimental data; (calc/exp in Å), **1pF**: 1.848/1.837(5); **3F**: 1.839/1.838(6). The largest difference was found for **1oF** (1.858/1.841(5), difference 0.017 Å, compare 3 standard deviations = 0.015 Å), although the angles describing the zigzag structure of this system were correctly reproduced. Globally, the optimizations using periodic lattice models reproduced correctly the intra- and intermolecular features of the halogen-bonded nickel–fluoride complexes.

### ^19^F NMR Chemical Shift Calculations in the Solid State

#### Periodic Lattice Models

The NMR chemical shifts and CST principal components for the nickel-bound fluorine in **1pF**, **1oF**, and **3F** were calculated under periodic boundary conditions using the GIPAW method (see the Computational Methods section for further information).^59^ As shown in Table 1, the isotropic chemical shifts (δ_iso_) are in good agreement with the experimental data, with a maximum deviation of 10 ppm. Moreover, the calculations reproduce the observed chemical shift of the fluoride in the order **1pF** > **1oF** > **3F**. At a qualitative level, the large anisotropy of the CSTs is correctly described, although at a quantitative level, the calculated δ_11_ and δ_22_ are too deshielded and δ_33_ too shielded relative to experiment. This led to calculated span (Ω) and skew (κ) parameter values that are larger than those observed experimentally (Table 1). Nonetheless, the trends in Ω and κ for the three complexes are correctly reproduced. Furthermore, the three components are attributed without ambiguity, with δ_11_, δ_22_, and δ_33_ around −150, −300, and −700 ppm, respectively.

**Table 1:** Experimental and Calculated ^19^F SSNMR CST Principal Components (in ppm) for **1pF**, **1oF**, and **3F** Using the Nonrelativistic GIPAW Method

	experimental δ (ppm)						calculated^a^ δ (ppm)					
complex	δ_iso_	δ_11_	δ_22_	δ_33_	Ω^b^	κ^c^	δ_iso_	δ_11_	δ_22_	δ_33_	Ω^b^	κ^c^
**1pF**	–359.8(2)	–165	–266	–645	480	0.58	–350.2	–135	–222	–694	558	0.69
**1oF**	–373.0(2)	–143	–302	–673	530	0.40	–371.5	–107	–270	–738	631	0.48
**3F**	–393.9(2)	–154	–298	–729	575	0.50	–393.4	–141	–233	–806	665	0.72

a Values calculated using the GIPAW method. See the Computational Methods section for more details.

b Ω calculated span of the chemical shift anisotropy, Ω=δ_11_-δ_33_.

c Calculated skew, κ = 3(δ_22_ – δ_iso_)/(δ_11_ – δ_33_).

#### Molecular Models

**Figure 3:**
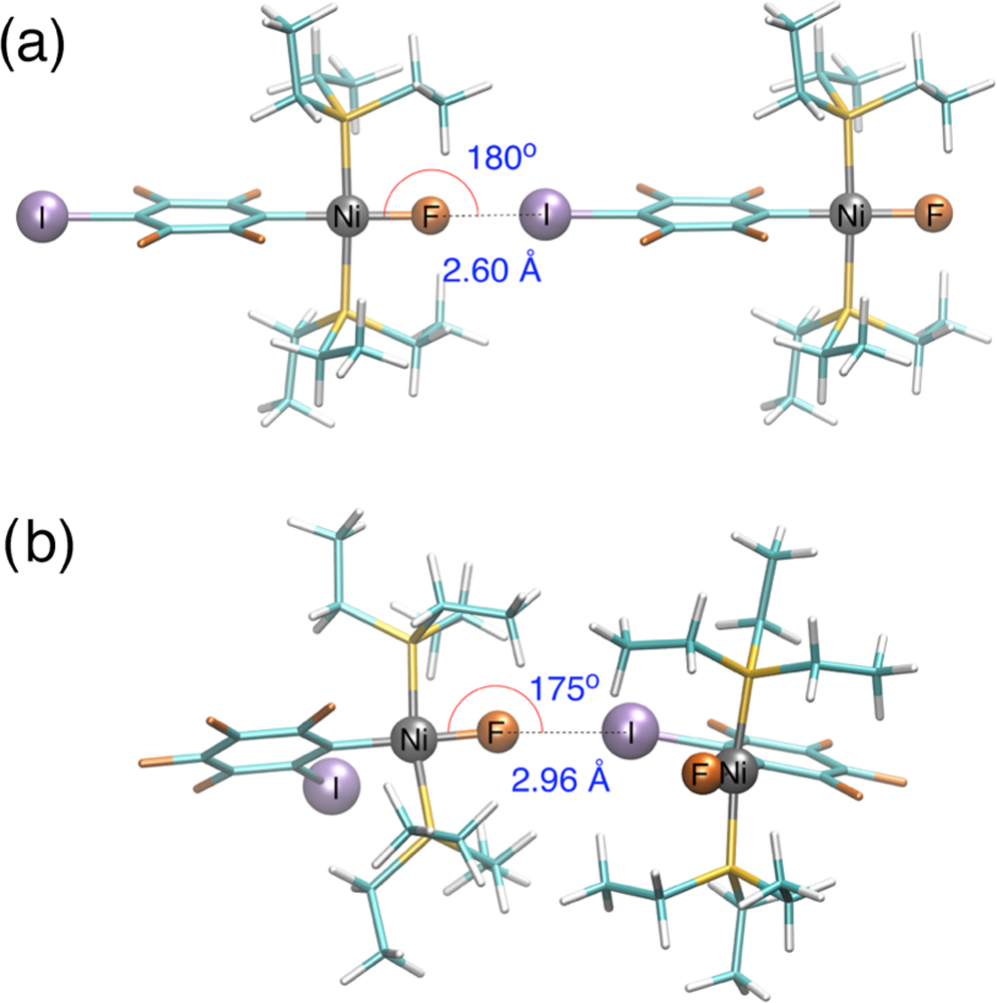
Dimer models (a) **1pF-d**_**(solid)**_ and (b) **1oF-d**_**(solid)**_ extracted from the structures optimized with periodic lattice models. The NiF···I(C) halogen bond is indicated by a dashed line. See Tables S4 and S5 for full structural information.

To estimate the influence of the crystal environment on the NMR signature of the nickel-bound fluoride, we studied molecular species having the same structure as in the crystal, but deprived of the crystal environment. Dimeric models of **1pF** and **1oF**, containing a single NiF···I(C) halogen bond, were thus extracted from the structures optimized with periodic lattice models and not further optimized. These species are labeled as **1pF-d**_**(solid)**_ and **1oF-d**_**(solid)**_ (Figure 3). The isotropic ^19^F NMR chemical shifts and CST principal components for **1pF-d**_**(solid)**_ and **1oF-d**_**(solid)**_ were calculated in the gas phase and compared with the values obtained from the periodic lattice models. In addition, we use these models for the analysis of the relativistic effects using both two- and four-component approaches (2c-ZORA and 4c-DKS) to represent the spin–orbit coupling (see the Computational Methods section for more details).

As shown in Table S7, all selected methods qualitatively capture the order of chemical shifts observed experimentally. The largest deviation was found for the nonrelativistic approach, with RMSD values of 53.0 (**1pF-d**_**(solid**)_) and 48.9 (**1oF-d**_**(solid)**_) ppm. We note that the inclusion of relativistic effects using two- or four-component approaches has little effect on the chemical shifts. The 2c-ZORA level showed RMSD values of 43.1 (**1pF-d**_**(solid**)_) and 39.4 ppm (**1oF-d**_**(solid)**_), while the 4c-DKS exhibited RMSD values of 36.8 (**1pF-d**_**(solid**)_) and 38.8 ppm (**1oF-d**_**(solid)**_). Hence, the 2c-ZORA level represents a good compromise, and the 4c-DKS gives the smallest deviations by a small margin. Furthermore, the largest systems (114 atoms) are accessible to the 2c-ZORA method, while the computational effort would be significant using 4c-DKS. In addition, the nonrelativistic calculations using either the periodic lattice models or the dimer models gave similar RMSD values and fairly good agreement with the observed values. Thus, intermolecular interactions in the crystal beyond the XB within the dimer have little effect on the NMR signatures. This allows us to use the dimeric models combined with the 2c-ZORA level for further analysis of the ^19^F SSNMR resonances.

### ^19^F NMR Chemical Shift Calculations for Solvated Complexes

The modeling of the ^19^F NMR chemical shifts in solution was carried out using the monomeric nickel–fluoride complexes where no XB is formed, named **1pF-m**_**(solv)**_, **1oF-m**_**(solv)**_, and **3F-m**_**(solv)**_. These species were optimized in benzene solution using the PBE0 functional and the implicit SMD solvation model^60^ (Table S9). The results show that the structural features of the complexes in solution are globally similar to those in the solid state. However, the Ni–F bond distance calculated in solution is significantly shorter (1.828 Å in **1pF-m**_**(solv)**_) than the one calculated with a periodic lattice model (1.848 Å in **1pF**). The C–I bond distance calculated in solution is also slightly shorter than in the solid state. In addition, the Ni–C(aryl) bond length for **1oF** is longer than in **1oF-m**_**(solv)**_, which could be due to some strain in the zigzag structure adopted in the solid state.

The ^19^F NMR chemical shifts in solution calculated at the 2c-ZORA-PBE/TZ2P level^61−68^ are in very good agreement with the experimental data, except for **1oF-m**_**(solv)**_ that was calculated to be ∼23 ppm more shielded than in the experiment (see Δδ, Table 2). The lack of inclusion of dynamics and explicit solvent effects on NMR could bias these results, especially in the case of **1oF-m**_**(solv)**_. For instance, the influence of the *ortho* iodine on the fluoride resonance, which could depend significantly on the libration of the aryl group, is unlikely to be properly represented by the single structure retained for the calculation. It should be noted that the calculated chemical shifts using nonrelativistic (NR) are within a few ppm of 2c-ZORA calculations, indicating again that relativity has little effect on the ^19^F NMR chemical shifts in these species.

**Table 2:** Experimental and Calculated (NR and 2c-ZORA) ^19^F NMR Chemical Shifts (δ) (in ppm) for **1pF-m**_**(solv)**_, **1oF-m**_**(solv)**_, and **3F-m**_**(solv)**_ in Benzene Solution

		calculations in benzene solution^c^			
complex	exp^a^	NR (δ)	Δδ^b^	2c-ZORA (δ)	Δδ^b^
**1pF-m**_**(solv)**_	–388.3	–390.5	–2.2	–388.4	–0.1
**1oF-m**_**(solv)**_	–397.9	–421.1	–23.2	–420.8	–22.9
**3F-m**_**(solv)**_	–394.3	–396.5	–2.2	–396.4	–2.1

a Values as reported in ref (39).

b Δδ = δ(calc) – δ(exp).

c See the Computational Methods section.

### Calculation of the NMR Signatures of the XB

In this work, we were particularly interested in an estimation of the NMR signatures of the XB for the nickel-bound fluoride, which is given by the difference in δ_iso_ between solid-state and solution NMR.^39^ Therefore, we analyzed the chemical shifts of the systems involved and not involved in the XB using the 2c-ZORA approach. The dimeric **1pF-d**_**(solid)**_ and **1oF-d**_**(solid)**_ species extracted from the structures optimized with periodic lattice models (Figure 3) were selected to represent the δ_iso_ in the solid state, while the monomers **X-m**_**(solv)**_ (**X** = **1pF**, **1oF**, and **3F**) optimized in benzene solution were selected to represent the δ_iso_ in solution. Additionally, we introduced here the corresponding monomeric species extracted also from the structures optimized with periodic lattice models, named **X-m**_**(solid)**_, where **X** = **1pF**, **1oF**, and **3F** (see Table S8 for full information on the calculated NMR resonances).

The observed and calculated ^19^F NMR signatures for **1pF**, **1oF**, and **3F** systems are shown in Table 3. The deshielding of the nickel-bonded fluorine in the presence of the XB and its order of magnitude is well reproduced for **1pF** (see Δδ_iso_, Table 3). Likewise, the results also nicely reproduce the fact that **3F** has essentially the same chemical shift in the solid state and in solution. On the other hand, a discrepancy appears in the NMR signature for **1oF** since the δ_iso_ of the monomer in solution (**1oF-m**_**(solv)**_) is not correctly reproduced (exp 24.9 vs calcd 47.5 ppm). Nevertheless, this chemical shift variation improves when comparing with **1oF-m**_**(solid)**_ (calcd 21.9 ppm). Using the monomeric species as reference also improve the agreement between experimental and calculated effects of the XB in the case of **1pF**. The sole mismatch in the case of **1oF** points out the possible importance of the structural features of the species in solution and the need for including dynamics and specific solvent effects, as already noted in previous NMR studies.^69−71^

**Table 3:** Experimental and Calculated (2c-ZORA) ^19^F NMR Chemical Shifts (in ppm) for the Halogen-Bonded Dimers **1pF-d**_**(solid)**_ and **1oF-d**_**(solid)**_, the Monomeric Species **X-m**_**(solid)**_, and the Monomers **X-m**_**(solv)**_ in Benzene Solution, Where **X** = **1pF**, **1oF**, and **3F**

	experimental δ_iso_			calculated δ_iso_ (molecular models)			calculated δ_iso_ (solution)	
**X**	solid state	solution	Δδ_iso_^a^	dimer **X-d**_**(solid)**_	monomer **X-m**_**(solid)**_	Δδ_iso_^b^	monomer **X-m**_**(solv)**_	Δδ_iso_^c^
**1pF**	–359.8(2)	–388.3	28.5	–356.7	–387.9	31.2	–388.4	31.7
**1oF**	–373.0(2)	–397.9	24.9	–373.3	–395.2	21.9	–420.8	47.5
**3F**	–393.9(2)	–394.3	0.4	NA^e^	–398.1	NA^e^	–396.4	–1.7^d^

a Calculated as Δδ_iso_ = δ_iso_ (solid) – δ_iso_ (solution).

b Calculated as the difference between **X-d**_**(solid)**_ and **X-m**_**(solid)**_.

c Calculated as the difference between **X-d**_**(solid)**_ and **X-m**_**(solv)**_.

d Calculated as the difference between **3F-m**_**(solid)**_ and **3F-m**_**(solv)**_.

e Not applicable.

#### Effect of the XB on the Chemical Shift Tensor

To understand the origin of deshielding at the nickel-bound fluoride upon XB, we compared the chemical shift tensors (CSTs) in **(1pF**/**1oF)-m**_**(solid)**_ and **(1pF**/**1oF)-d**_**(solid)**_. In this way, this deshielding can be exclusively attributed to the XB interaction. For comparison, **3F-m**_**(solid)**_ has been included as a representative species that does not become involved in a XB. The calculated principal components and the orientation of the principal axes of the ^19^F SSNMR CST are shown in Table 4 for monomers and dimers. All species have identical orientations of the principal axes of the CST and identical ranking of the principal components: the most deshielded component (δ_11_) is along Ni–F, while the most shielded one (δ_33_) is perpendicular to the Ni coordination plane. This pattern is characteristic of a square-planar Ni^II^–fluoride complex as can be established by comparison with a model system NiH(F)(PH_3_)_2_. The calculated CST values for this species are consistent: same directions of the principal axes and (δ_11_ > δ_22_ ≫ δ_33_) in similar regions of the spectra (see Table S11).

**Table 4:**
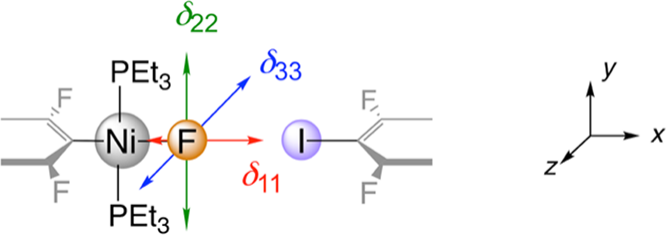
Calculated (2c-ZORA) ^19^F SSNMR CST Principal Components (in ppm) of **1pF-d**_**(solid)**_, **1oF-d**_**(solid)**_, and **3F-m**_**(solid)**_^a^

	calculated δ (ppm)			
species	δ_iso_	δ_11_	δ_22_	δ_33_
**1pF-d**_**(solid**)_	–356.7	–143.7	–223.7	–702.8
**1pF-m**_**(solid**)_	–388.0	–101.7	–245.6	–816.7
**Δδ**_**ii**_^**d-m**^	31.3	–42.0	21.9	113.9
**1oF-d**_**(solid**)_	–373.3	–118.9	–271.9	–729.2
**1oF-m**_**(solid**)_	–395.2	–97.4	–288.8	–799.4
**Δδ**_**ii**_^**d-m**^	21.9	–21.5	16.9	70.2
**3F-m**_**(solid)**_	–398.2	–152.1	–234.4	–808.0

a The orientations of the principal axes of the CST are shown at the top with red, green, and blue arrows corresponding to δ_11_, δ_22_, and δ_33_. The experimental values are given in Table 1.

**Figure 4:**
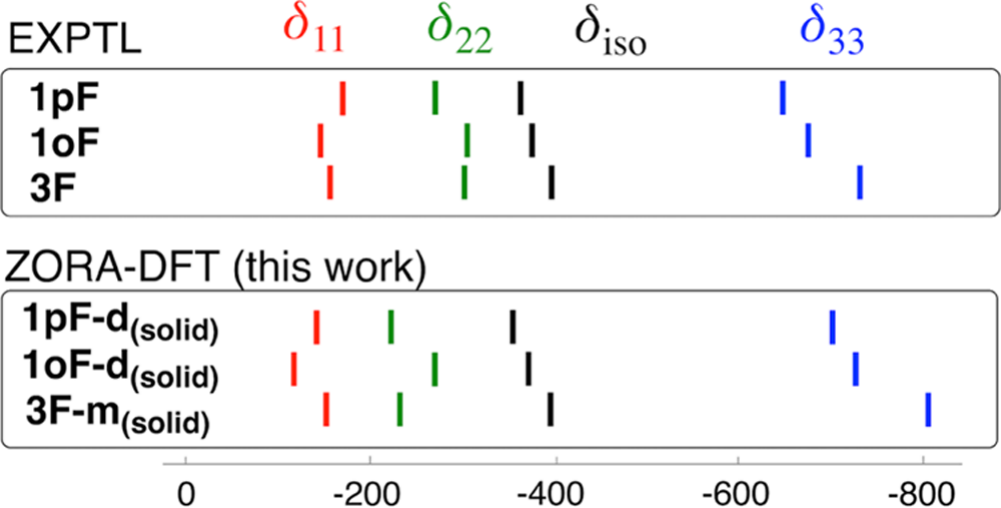
Positions of the ^19^F SSNMR chemical shift tensor components (in ppm) for (top) experimental **1pF**, **1oF**, and **3F**(39) and (bottom) 2c-ZORA calculations for **1pF-d**_**(solid)**_, **1oF-d**_**(solid)**_, and **3F-m**_**(solid)**_.

The positions of the resonances for the calculated molecular models and the observed crystalline systems are in excellent agreement, as illustrated in Figure 4. The comparison is especially satisfying for δ_33_, with almost identical observed and calculated variations for **1pF**, **1oF**, and **3F**. The results are also very good for δ_11_, but some deviation from the experimental values is found in δ_22_, especially for **1oF-d**_**(solid)**_. For **1pF**, this work gives results which are more accurate than in the earlier study,^39^ although the observed pattern for this system was already reproduced using a model complex of **1pF** halogen-bonded to IC_6_F_5_.

The effect of the XB is different for each component: δ_11_ is shielded by the XB by less than 50 ppm; in contrast, δ_22_ and δ_33_ are both deshielded, the main effect being found for δ_33_ with Δδ_33_^d-m^ of 114 (**1pF**) and 70 (**1oF**) ppm (see Δδ_ii_^d-m^, Table 4). Thus, the deshielding of δ_iso_ with the XB is due to the change of the two principal components that are associated with the principal axes perpendicular to the Ni–F bond, and mainly with the one that is perpendicular to the coordination plane of the complex. Similarly, a simulation of the effect of XB in HF···ICH_3_ also indicated a deshielding of the components perpendicular to the H–F axis (Table S11 and Figure S2).

### Analysis of the ^19^F NMR Shielding Components

To understand the nature of the CST and its variation upon XB, we considered a simple method for examining the relationship between MOs and CST, based on Ramsey’s perturbation equations of nuclear shielding^44,47^ (see the Supporting Information for further details). This analysis method has been used successfully for main group atoms of nuclear spin of 1/2, such as carbon, nitrogen, and phosphorus.^43,44,47,50−52^ In the following, we use the shielding, σ, rather than chemical shift, δ, since it is the former that is calculated and analyzed. This corresponds to a change of reference (δ = σ_ref_ – σ) where σ_ref_ is the ^19^F shielding in CFCl_3_ (see the Computational Methods section). In a 2c-ZORA scheme, the shielding can be split into diamagnetic (σ^dia^) and paramagnetic plus spin–orbit (SO) (σ^p+SO^) contributions. For *n* = 2 and 3 main group elements, the diamagnetic contribution is usually almost isotropic.^39−44^ This is less the case for fluorine in the present complexes, where σ_11_^dia^ is about 40 ppm more shielding than σ_22_^dia^ and σ_33_^dia^ (Table 5). However, the differences between σ_11_, σ_22_, and σ_33_ are much larger in the σ^p+SO^ terms, as illustrated by the difference of over 700 ppm between σ_11_^p+SO^ and σ_33_^p+SO^ for both **1pF-m**_**(solid)**_ and **1oF-m**_**(solid)**_ systems. Thus, as was found for other nuclei such as carbon,^72−78^ it is sufficient to consider only the σ^p+SO^ term to probe the origin of the anisotropy of the shielding tensor. Furthermore, the spin–orbit part in σ^p+SO^ is small as previously shown by the equivalence in the chemical shifts calculated with NR and 2c-ZORA methods. It is thus possible to focus on the paramagnetic part of the shielding.

**Table 5:** Calculated (2c-ZORA) Shielding Tensor Principal Components for the Monomeric and Dimeric Species, **X-m**_**(solid)**_ and **X-d**_**(solid)**_, with Diamagnetic σ_*ii*_^dia^ and Paramagnetic + Spin–Orbit Contributions σ_ii_^p+SO^ (in ppm)

species	σ_iso_	σ_11_	σ_22_	σ_33_	σ_11_^dia^	σ_22_^dia^	σ_33_^dia^	σ_11_^p+SO^	σ_22_^p+SO^	σ_33_^p+SO^
**1pF-m**_**(solid)**_	537.6	251.3	395.2	966.3	482.8	455.3	446.6	–231.4	–60.2	519.7
**1pF-d**_**(solid)**_	506.3	293.3	373.3	852.4	494.8	455.9	447.7	–201.4	–82.6	404.5
**Δσ**_***ii***_^**d-m**^	–31.3	42.0	–21.9	–113.9	12.0	0.6	1.1	30.0	–22.4	–115.2
**1oF-m**_**(solid)**_	544.8	247.0	438.4	949.0	481.2	455.3	446.7	–234.1	–16.9	502.2
**1oF-d**_**(solid)**_	522.9	268.5	421.5	878.8	489.2	453.9	444.9	–220.7	–32.4	433.9
**Δσ**_***ii***_^**d-m**^	–21.9	21.5	–16.9	–70.2	8.0	–1.4	–1.8	13.4	–15.5	–68.3

We now analyze the σ_ii_^p+SO^ terms. To interpret these terms, Zilm et al. used an orbital “rotation” model in the case of atomic p orbitals and stated that “The paramagnetic terms arise from the interaction of occupied orbitals with virtual orbitals that are rotated by 90° and may lead to either deshielding or shielding. The normal interaction leads to deshielding.”^79^ In this work, it is helpful to consider first the cases of H–F and Cl–F, before presenting the nickel–fluoride complexes.

**Scheme 2:**
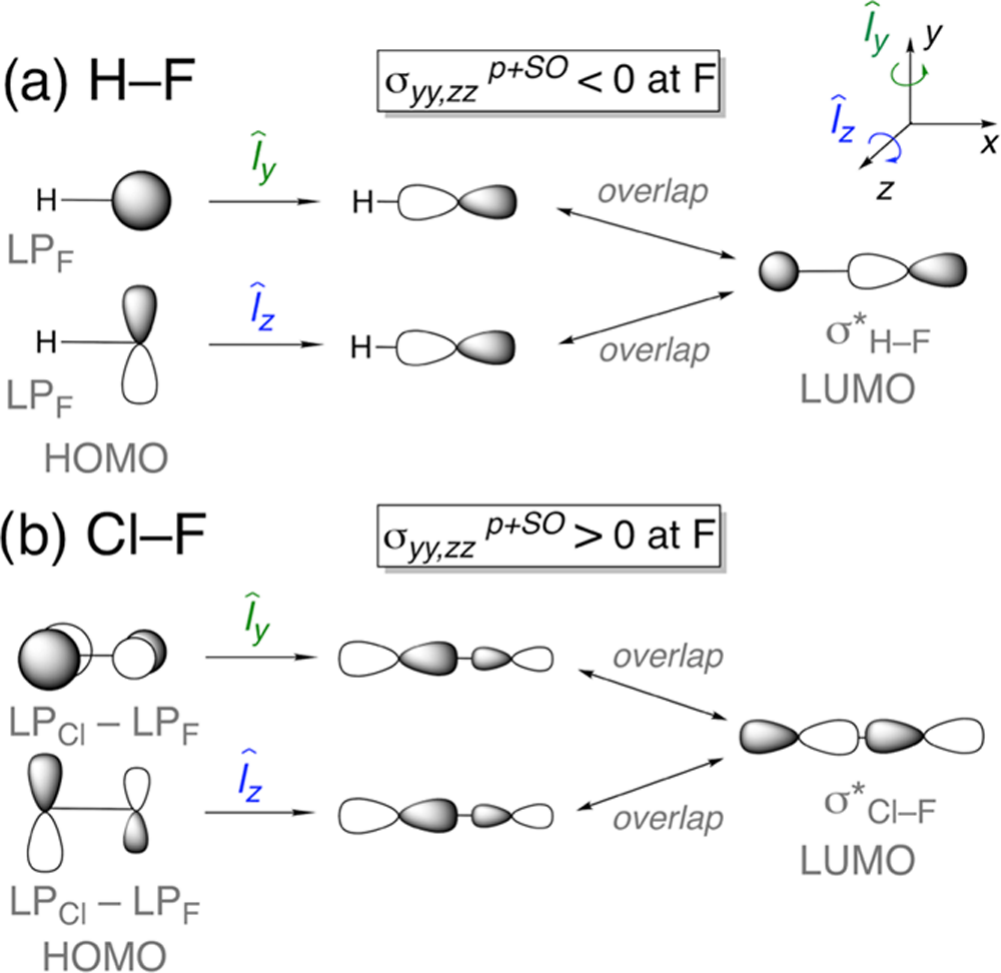
Illustration of the Sign of the Paramagnetic Coupling of Occupied to Vacant Orbitals at F Using the Orbital Rotation Model Applied to the Representative Cases (a) H–F and (b) Cl–F;^79^ All Orbitals Are Drawn Schematically with a Focus on Their Topology

In H–F, which is aligned with the *x* axis, the large negative (deshielding) paramagnetic terms in the direction perpendicular to H–F (*y* or *z*) can be traced back to contributions from the paramagnetic coupling of the F 2p*_z_* (or 2p*_y_*) lone pairs (LPs) with the vacant σ_H–F_^*^ MO via the *l̂_y_* (or *l̂_z_*) angular momentum operator, as shown in Scheme 2a (see the Supporting Information for further details). In contrast, there is no vacant orbital to couple paramagnetically with either of these F 2p*_y_* or 2p*_z_* LPs via the *l̂_x_* angular momentum operator. Consequently, the most shielded term is along H–F and the directions perpendicular to H–F are deshielded.

In Cl–F, the F is shielded relative to the anionic F^–^.^80^ Cornwell showed that this arises from the magnetic interaction of the out-of-phase combination of Cl 3p and F 2p LPs with the vacant σ_Cl–F_^*^ orbital.^81^ As part of their general interpretation, Zilm et al. pointed out the effect of p orbitals on neighboring atoms: “when two p orbitals in an occupied MO have opposite phase, the dominant interaction will lead to deshielding but the other atom will be shielded.” Thus, in Cl–F, Cl is deshielded and F shielded.^79^ The occupied and vacant MOs involved in the paramagnetic shielding at F are shown schematically in Scheme 2b. Since the only vacant orbital is σ_Cl–F_^*^, there is no paramagnetic term along the Cl–F direction. As a result, the most shielded directions for F in Cl–F are perpendicular to the molecular axis (see the Supporting Information for further details). Related findings have been obtained to account for the effect of substituents in a set of aliphatic fluorides.^82^

#### Case of the Square-Planar Nickel–Fluoride Monomer

**Scheme 3:**
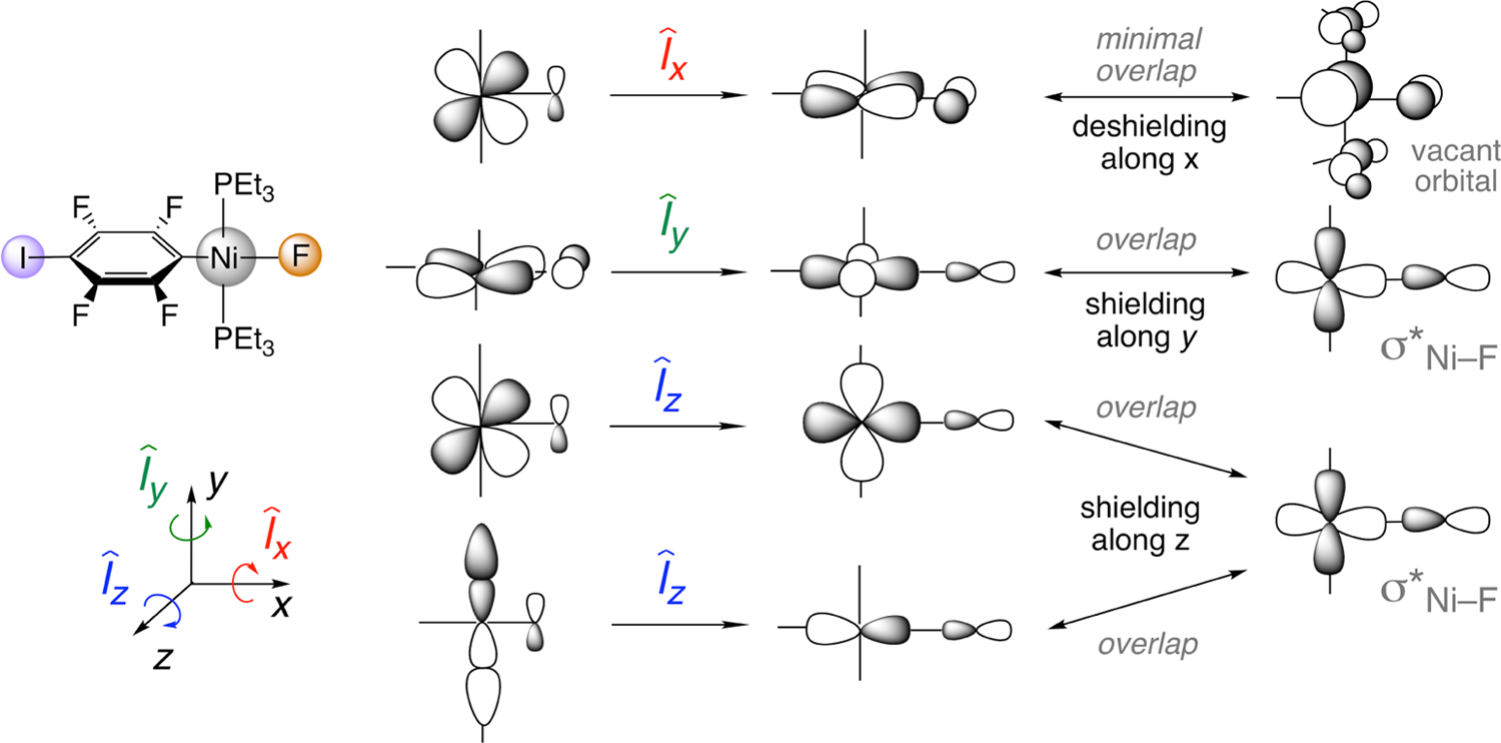
Coupling of Occupied Ni (or Ni-Phosphine)/Fluorine 2p Lone Pairs *via* the Angular Momentum Operator and Their Contribution to the Paramagnetic Term in the *trans*-[NiF(2,3,5,6-C_6_F_4_I)(PEt_3_)_2_] Complex All orbitals are drawn schematically with a focus on their topology.

A similar analysis to that for Cl–F can be applied to the nickel–fluoride complexes, keeping in mind that the orbital rotation model applied to the metal d orbitals does not always correspond to 90° rotation.^83^ In the present case, 45 and 90° rotations will be used; a full description of the action of angular momentum operators on all d orbitals can be found in ref (46). As shown in Scheme 3, the fluorine 2p*_y_* and 2p*_z_* LPs perpendicular to the Ni–F axis are engaged in four-electron interaction with occupied Ni (3d*_xy_* and 3d*_xz_*) and Ni–P orbitals. Consequently, the out-of-phase combinations of these Ni 3d (or Ni–P bonding) and symmetry-adapted F 2p orbitals are occupied and all out-of-phase occupied orbitals have larger coefficients on Ni (or Ni–P) than on F due to the high electronegativity of fluorine. Thus, in all cases, the coupling of these occupied orbitals with the vacant σ_Ni–F_^*^ orbital via the appropriate angular momentum operator (*l̂_z_* or *l̂_y_*) leads to shielding at the fluorine. Note that the action of *l̂_z_* or *l̂_y_* on Ni (3d*_xy_* or 3d*_xz_*) gives Ni (3d_*x*^2^–*y*^2^_ or 3d_*z*^2^–*x*^2^_) atomic functions, respectively. Both of the orbitals overlap significantly with σ_Ni–F_^*^, which has a strong Ni 3d_*x*^2^–*y*^2^_ character.^46^ This accounts for the large shielding influence of Ni at F in the *z* (δ_33_) and *y* (δ_22_) directions, respectively. Furthermore, there are more occupied orbitals (with Ni center and the Ni–P bonding character) in the *xy* plane of the square-planar complex than in the perpendicular *xz* plane. Therefore, fluorine is more shielded perpendicular to the Ni coordination plane, i.e., along the *z* axis (δ_33_). In contrast, the shielding influence of Ni at F along *x* (the Ni–F direction) is almost nil. Indeed, the paramagnetic term along *x* comes in good part from the coupling via the *l̂_x_* angular momentum operator of the occupied Ni 3d*_xy_* – F 2p*_y_* orbital with a high-lying vacant orbital having large Ni 4p*_z_* and σ_P–C_^*^ and small F 2p*_z_* characters (Scheme 3).^84^ The action of *l̂_x_* on Ni 3d*_xy_* gives Ni 3d*_xz_*,^46^ and thus the overlap at Ni between the rotated occupied and the vacant orbital is nil. Only a fluorine deshielding contribution remains, but it is small because the vacant orbital is at high energy. An NLMO analysis^45,46^ supports the presented qualitative orbital rotation model (Table S13).

As a consequence of these various effects, the most deshielded direction is along Ni–F and the directions perpendicular to Ni–F are shielded especially the one perpendicular to the coordination plane. A similar interpretation of the shielding role of the occupied d orbitals of the metal was given for interpreting the isotropic ^19^F chemical shift in a cobalt–fluorine complex.^41^

### Case of the Halogen-Bonded Nickel–Fluoride Dimer

To understand the effect of the XB on the NMR spectra, we first carried out an energy decomposition analysis of the self-assembled nickel complexes considering **X-d**_**(solid)**_ and **X-m**_**(solid)**_ (**X** = **1pF** and **1oF**) to obtain some information on the energetics. We use the variational EDA^85,86^ based on absolute localized molecular orbitals^87,88^ (ALMO-EDA), which reveals that the interactions between these neutral inorganic species are qualitatively similar to that found for the charged halogen-bonded organic species CX_3_I···Y^–^ (X = F, Cl, Br and I; Y = F, Cl and Br).^14^ The noticeable result is that the total interaction energy is 17.7 and 8.7 kJ mol^–1^ for **1pF** and **1oF**, respectively, suggesting that the neutral nickel-bonded fluoride is a good XB acceptor toward the C–I bond of the iodoaryl ligand acting as XB donor. Although no experimental values of these interaction energies in the solid are available, they are of the expected order of magnitude for XBs. The ALMO-EDA study shows that the largest attractive term derives from the permanent electrostatics but that it is offset by the even larger Pauli repulsion. Thus, in these neutral nickel complexes, the stabilizing polarization and charge transfer terms contribute together with permanent electrostatics to the attraction between the two monomeric complexes (see Table S12 for further details).

We now turn our attention to the characteristics of the ^19^F NMR shielding tensor in **1pF-d**_**(solid)**_ and **1oF-d**_**(solid)**_, relative to that in **1pF-m**_**(solid)**_ and **1oF-m**_**(solid)**_, respectively (Table 5). As we mentioned before, the major changes between the halogen-bonded dimer and the corresponding monomer are mostly contained in a deshielding of the σ_33_ component (Tables S13 and S14). We recall that the paramagnetic term is inversely proportional to the energy gap between the occupied and vacant orbitals linked by the angular momentum operator. Our calculations show an increase in the energy gap in the dimer relative to the monomer for the main pair of MOs involved in the shielding, notably Ni 3d*_xy_* – F 2p*_y_* and σ_Ni–F_^*^ (Scheme 3). This change allows a simple interpretation of the decrease of the paramagnetic term σ_33_^p+SO^, and consequent reduction in shielding. The lowering of the energy of the occupied orbital upon formation of the XB is a direct consequence of the bonding interaction between the two systems. It should be noted though that the bonding interaction involves mostly orbitals along the Ni–F and I–C bonds, but lowering of other orbitals is also expected. The lowering of the lowest unoccupied molecular orbital (LUMO) is more difficult to estimate but is systematically seen in all models of halogen-bonded systems considered. Further studies with a wider set of metal–fluoride complexes^89^ and nonmetallic fluorine species are currently underway to extend the interpretation presented in this work. In addition, it is not yet possible to elaborate on the existence or lack of relationship between the energetics of the XB and its NMR signature. For this, we also need to study a larger set of systems. One can however remark that mostly orbitals co-axial with the XB are involved in its strength, while NMR shielding is based on the contribution of occupied orbitals perpendicular to the XB direction and empty co-axial orbitals. Since different orbitals are involved in the two phenomena, the nature of relationship cannot be established from this work.

## Conclusions

The effect of the XB on the ^19^F SSNMR chemical shift was demonstrated in a previous study by comparing the NMR spectra of *trans*-[NiF(2,3,5,6-C_6_F_4_I)(PEt_3_)_2_] **1pF** and *trans*-[NiF(2,3,4,5-C_6_F_4_I)(PEt_3_)_2_] **1oF** complexes,^39^ with that of a complex incapable of XB formation, *trans*-[NiF(C_6_F_5_)(PEt_3_)_2_] **3F**.^42^ For **1pF** and **1oF**, the presence of the XB is manifested through deshielding of the nickel-bound ^19^F SSNMR isotropic chemical shifts (δ_iso_), relative to values obtained in solution, where no XB is observed.^39^ The present work has used a computational protocol to provide insight into the specificities of the ^19^F chemical shift resonances in square-planar nickel–fluoride complexes involved and not involved in a XB situation.

The average chemical shift δ_iso_ and also the ranking of the principal components δ_11_, δ_22_, and the strongly shielded δ_33_ are well reproduced for the nickel-bonded fluoride with 2c-ZORA calculations for a halogen-bonded dimer model. This dimer model is extracted from the structures optimized with periodic lattice models, which themselves are in good agreement with experiment. This result indicates that the XB has a significant effect but the crystal packing has no visible influence on the fluoride chemical shifts of these complexes. The analysis of relativistic effects also indicates that the ^19^F NMR resonances are well reproduced with the 2c-ZORA Hamiltonian, although agreement improves with the four-component Dirac–Kohn–Sham approach. In general, the agreement with experiment is better for **1pF** than for **1oF**.

Comparison between the calculated ^19^F chemical shifts (isotropic and principal components) of the isolated monomeric square-planar complexes and the corresponding halogen-bonded dimers provides the change in the NMR resonances due to the XB. These calculated shifts are in good agreement with the observed values, evaluated as the differences between the resonances in solution, where XB is absent and that in the solid state, where it is present. In particular, the calculations reproduce nicely the downfield shift of δ_iso_ on forming the XB driven mostly by the change in δ_33_ with an additional contribution from δ_22_.

We used the orbital rotation model to analyze the paramagnetic terms of three principal components of the ^19^F CST in the monomeric nickel–fluoride complex. The paramagnetic terms, come from the coupling *via* the angular momentum operator of occupied and vacant orbitals. In these square-planar complexes, they involve the F 2p lone pairs combined in an antibonding way with the symmetry-adapted Ni and Ni–P occupied orbitals and the vacant σ_Ni–F_^*^ orbital, essentially the LUMO (Ni d_*x*^2^–*y*^2^_). As exemplified by Zilm et al. for the emblematic Cl–F,^79^ the paramagnetic term is positive, and thus shielding increases at fluorine in the directions perpendicular to the axis since the Cl/F out-of-phase highest occupied orbital is dominated by chlorine. In the nickel complexes, the Ni or Ni–P character dominates in the Ni/F out-of-phase highest occupied orbitals and thus induces a shielding at fluorine in the direction perpendicular to the Ni–F axis. Since there are more Ni/F occupied antibonding orbitals in the coordination plane than out of it, the most shielded direction is perpendicular to the coordination plane. The least shielded direction δ_11_, which is along Ni–F, involves occupied and vacant orbitals perpendicular to the Ni–F axis. However, the Ni shielding contribution at F is essentially nil because of the lack of overlap at Ni between the two coupled orbitals which have 3d and 4p characters, respectively. We show that these features are characteristic of square-planar nickel–fluoride complexes.

In the halogen-bonded nickel–fluoride systems, the positive paramagnetic terms at F in the δ_22_ and δ_33_ components are decreased. A rationale for this is that the occupied-vacant energy gap between orbitals involved in the shielding increases on forming the XB as a direct consequence of the bonding interaction. The generality of this interpretation needs to be assessed by the study of a wider range of metal-bound fluoride complexes.

## Computational Methods

### Structure Optimization

#### Calculations with Periodic Lattice Models

**1pF**, **1oF**, and **3F** were optimized at the density-functional theory (DFT) level using periodic boundary conditions, as implemented in *CRYSTAL-17*.^90,91^ The X-ray diffraction data^39,42^ were employed to generate the initial unit-cell parameters and atomic positions. The experimentally observed symmetry of the crystal lattices has been maintained during structural optimizations. The performance of the PBE0^92^ and HSE06^93^ functionals and the inclusion of Grimme’s dispersion D3 correction^57^ in reproducing the solid-state structures was assessed. Stuttgart–Dresden relativistic pseudopotentials with the associated Gaussian-type basis sets were employed for Ni, I, and P; the pob_TZVP_rev2^58^ basis set for the rest of the atoms (F, C, H). Calculations used a Monkhorst–Pack *k*-points mesh of 6 × 4 × 4 (**1pF**), 4 × 4 × 4 (**1oF**), and 4 × 4 × 2 (**3F**). It appeared that the inclusion of dispersion D3 correction was not beneficial to the reproduction of structural parameters that are important for the NMR shielding calculations, even if it was beneficial for the unit-cell parameters. The PBE0 functional that best reproduced features essential to the NMR study was thus selected.

#### Calculations for Solvated Complexes

The three monomeric nickel–fluoride complexes, named **1pF-m**_**(solv)**_, **1oF- m**_**(solv)**_, and **3F-m**_**(solv)**_, were optimized using the *Gaussian* software,^94^ including the implicit SMD solvation model^60^ for benzene. These calculations were carried out using the PBE0 functional combining a Stuttgart–Dresden relativistic pseudopotential with its associated basis set for Ni, the aug-pcSseg-2^95^ basis set for F, and pcseg-2^96^ basis sets for the rest of the atoms.

### ^19^F NMR Chemical Shift Calculations in the Solid State

#### Periodic Lattice Models

The NMR chemical shifts and chemical shift tensor components for the nickel-bound fluorine in **1pF**, **1oF**, and **3F** were computed with the gauge-including projector augmented wave (GIPAW) method,^59^ as implemented in the *Quantum Espresso*(97) software (ver. 7.1). Calculations used a Monkhorst–Pack *k*-point grid of 6 × 4 × 4 (**1pF**), 4 × 4 × 4 (**1oF**), or 4 × 4 × 2 (**3F**) and a cutoff value for energy was set to 90 Ry.

#### Molecular Models

The dimeric (**1pF-d**_**(solid)**_ and **1oF-d**_**(solid)**_) and monomeric (**1pF-m**_**(solid)**_, **1oF-m**_**(solid)**_, and **3F-m**_**(solid)**_) models were extracted from the optimized crystalline systems, without performing any further geometry optimization. The ^19^F NMR chemical shifts were calculated in gas phase using the PBE functional^64,65^ in conjunction with the all-electron Slater-type orbitals (STO) TZ2P basis sets.^67^ The calculations were performed including scalar (SR) and spin–orbit (SO) relativistic effects at the two-component (2c) level using the zeroth-order regular approximation (ZORA) Hamiltonian,^61−63,66,68^ as implemented in the *ADF* program.^98,99^ The gauge-origin dependence was handled using gauge-including atomic orbitals (GIAO) approach.^100^

The 2c-ZORA results for **1pF-d**_**(solid)**_ and **1oF-d**_**(solid)**_ were compared with those obtained by a nonrelativistic (NR) method using *Gaussian* software.^94^ The PBE functional was selected, combining a Stuttgart–Dresden relativistic pseudopotential with its associated basis set for Ni, the aug-pcSseg2 basis set for F and pcseg-2 basis sets for the rest of the atoms. Additional calculations were performed using the fully four-component Dirac–Kohn–Sham (4c-DKS) method in combination with the Dirac–Coulomb Hamiltonian,^101,102^ as implemented in the *ReSpect* program.^103^ The PBE functional was selected combining the all-electron Dyall’s VTZ basis sets for Ni, F, and I, and the uncontracted pcS-2^104,105^ basis sets for the rest of the atoms. Note that although some minor differences between the NR-PBE, 2c-ZORA, and 4c-DKS approaches can be expected, for example, from the different types of basis sets (Gaussian vs Slater basis sets), these calculations can be used to evaluate the importance of relativistic effects.

### ^19^F NMR Chemical Shift Calculations for Solvated Complexes

The ^19^F NMR chemical shifts of the monomers in solution (**1pF-m**_**(solv)**_, **1oF-m**_**(solv)**_, and **3F-m**_**(solv)**_) were calculated using the geometries optimized with the SMD solvation model^60^ for benzene. These calculations were carried out using the COSMO model for simulating bulk solvation in benzene at the 2c-ZORA-PBE/TZ2P level, implemented in the *ADF* program. The 2c-ZORA results were compared with those obtained by a nonrelativistic (NR) method including the SMD model for solvation in benzene, as implemented in *Gaussian* software. These calculations were obtained using the PBE functional combining a Stuttgart–Dresden relativistic pseudopotential with its associated basis set for Ni, the aug-pcSseg2 basis set for F and pcseg-2 basis sets for the rest of the atoms. All ^19^F NMR chemical shifts (δ_iso_) are referenced to trichlorofluoromethane (CFCl_3_, calculated shielding = 139.0 (NR), 149.6 ppm (2c-ZORA)).

### ^19^F NMR Chemical Shift Calculations for H–F, HF···ICH_3_, and NiH(F)(PH_3_)_2_ Species

These species were optimized at the ZORA-PBE0/TZ2P level. Subsequent ^19^F NMR chemical shift calculations were carried out at the ZORA-PBE/TZ2P level. For HF···ICH_3_, the XB distance was set equal to that in **1pF-d**_**(solid)**_ (2.657 Å), while the rest of the structural parameters were optimized.

### Analysis of the NMR Chemical Shifts and Electronic Structure

Analyses of the bonding and the shielding were carried out with the *NBO 6.0* program.^106,107^ Interaction between **X-m**_**(solid)**_ to yield **X-d**_**(solid)**_ for **X** = **1pF** and **1oF** was evaluated with the variational EDA^85,86^ based on absolute localized molecular orbitals^87,88^ (ALMO-EDA) approach of Head-Gordon, as implemented in the *Q-Chem 5.2* software package.^108^ For consistency, the same combination of functional and basis set was used for the NBO and NMR calculations.

A dataset collection of the computational results is available in the *ioChem-BD* repository^109^ and can be accessed via https://doi.org/10.19061/iochem-bd-6-189.

## Supporting Information Available

Additional computational data and figures (PDF)
Cartesian coordinates (ZIP)
