# Supplementary material for: Solid-State 19F NMR Chemical Shift in Square-Planar Nickel–Fluoride Complexes Linked by Halogen Bonds
Source: Inorg Chem. 2023 Mar 15;62(12):4835–46. doi: 10.1021/acs.inorgchem.2c04063 (PMC10052355; doi:10.1021/acs.inorgchem.2c04063)
Supplement: Supplementary file 1 — ic2c04063_si_001.pdf [file ic2c04063_si_001.pdf]

Supporting Information for

Solid-state ^{19}F NMR chemical shift in square-planar nickel-fluoride complexes linked by halogen bonds

Abril C. Castro,[a] Michele Cascella,[a] Robin N. Perutz,[b] Christophe Raynaud*,[c] and Odile Eisenstein* [a, c]*

[a]Hylleraas Centre for Quantum Molecular Sciences, Department of Chemistry, University of Oslo, 0315 Oslo, Norway

[b]Department of Chemistry, University of York, Heslington, York, YO10 5DD, United Kingdom

[c]ICGM, Université Montpellier, CNRS, ENSCM, 34090 Montpellier, France

Corresponding Authors

*Abril C. Castro; e-mail: abril.castro@kjemi.uio.no

*Christophe Raynaud; e-mail: christophe.raynaud1@umontpellier.fr

*Odile Eisenstein; e-mail: odile.eisenstein@umontpellier.fr

CONTENTS

Table S1. Experimental and calculated unit-cell parameters for **1pF** (monoclinic cell, I2 space group) with periodic boundary conditions (PBC) as a function of the functional. S4

Table S2. Experimental and calculated unit-cell parameters for **1oF** (monoclinic cell, $P2_1$ space group) with periodic boundary conditions (PBC) as a function of the functional. S5

Table S3. Experimental and calculated unit-cell parameters for **3F** (orthorhombic cell, $P2_12_12_1$ space group) with periodic boundary conditions (PBC) as a function of the functional. S6

Table S4. Experimental and optimized structure, selected bond distances (in Å) and angles (in degrees), for **1pF** with periodic boundary conditions (PBC) as a function of the functional. S7

Table S5. Experimental and optimized structure, selected bond distances (in Å) and angles (in degrees), for **1oF** with periodic boundary conditions (PBC) as a function of the functional. S8

Table S6. Experimental and optimized structure, selected bond distances (in Å) and angles (in degrees), for **3F** with periodic boundary conditions (PBC) as a function of the functional. S9

Table S7. Calculated ^{19}F NMR shielding ($\boldsymbol{\sigma}$) and chemical shift ($\boldsymbol{\delta}$) tensor components (in ppm) for **1pF-d$_{(solid)}$** and **1oF-d$_{(solid)}$** dimers. S9

Table S8. Calculated ^{19}F NMR shielding ($\boldsymbol{\sigma}$) and chemical shift ($\boldsymbol{\delta}$) tensor components (in ppm) of **1pF-m$_{(solid)}$**, **1oF-m$_{(solid)}$**, and **3F-m$_{(solid)}$**. S10

Table S9. Selected bond distances (in Å) and angles (in degrees) for **1pF-m$_{(solv)}$**, **1oF-m$_{(solv)}$**, and **3F-m$_{(solv)}$** optimized in benzene solution using the SMD method. S10

Table S10. Calculated ^{19}F NMR shielding ($\boldsymbol{\sigma}$) and chemical shift ($\boldsymbol{\delta}$) tensor components (in ppm) for **1pF-m$_{(solv)}$**, **1oF-m$_{(solv)}$**, and **3F-m$_{(solv)}$** in benzene solution. S11

Table S11. Calculated shielding tensor principal components of fluorine with diamagnetic and paramagnetic plus spin-orbit contributions (in ppm) for the selected Ni–F complexes and HF···I–CH_3 model. Values calculated at the 2c-ZORA-PBE/TZ2P level. S11

Table S12. ALMO-EDA analysis (in kJ/mol) for **1pF-d$_{(solid)}$** and **1oF-d$_{(solid)}$** considering the dimers in terms of the two monomeric units, **1pF-m$_{(solid)}$** and **1oF-m$_{(solid)}$**, computed in gas-phase at the PBE/TZ2P level. S12

Table S13. Shielding tensor components ($\boldsymbol{\sigma^{p+SO}}$) in **1pF-m$_{(solid)}$** and **1pF-d$_{(solid)}$** using VB representation of the systems where Ni forms covalent bonds with fluorine and one of the phosphine ligands. S13

Table S14. Shielding tensor components in **1oF-m$_{(solid)}$** and **1oF-d$_{(solid)}$**. The valence representation is similar to that for **1pF**. S14

Table S15. NBO analysis of selected canonical molecular orbitals for **1pF-m$_{(solid)}$**. S15

Table S16. NBO analysis of selected canonical molecular orbitals for **1pF-d$_{(solid)}$**. S17

Table S17. Canonical molecular orbitals of H–F model structure. S19

Table S18. NBO analysis of selected canonical molecular orbitals for HF···ICH_3 model structure S19

Relationship between chemical shift and frontier molecular orbitals S21

Orbital Rotation Model approach S22

Figure S1. Schematic description of paramagnetic shielding ($\boldsymbol{\sigma}$, ppm) for the case of H–F molecule.. .... S22

Figure S2. HF···ICH_3 model. The F···I distance associated with the XB bond was set equal to that in **1pF-d** and the rest of structural parameters were optimized at the PBE0/TZ2P level. S23

Analysis of the shielding components in Cl–F S24

Table S1. Experimental and calculated unit-cell parameters for **1pF** (monoclinic cell, I2 space group) with periodic boundary conditions (PBC) as a function of the functional.

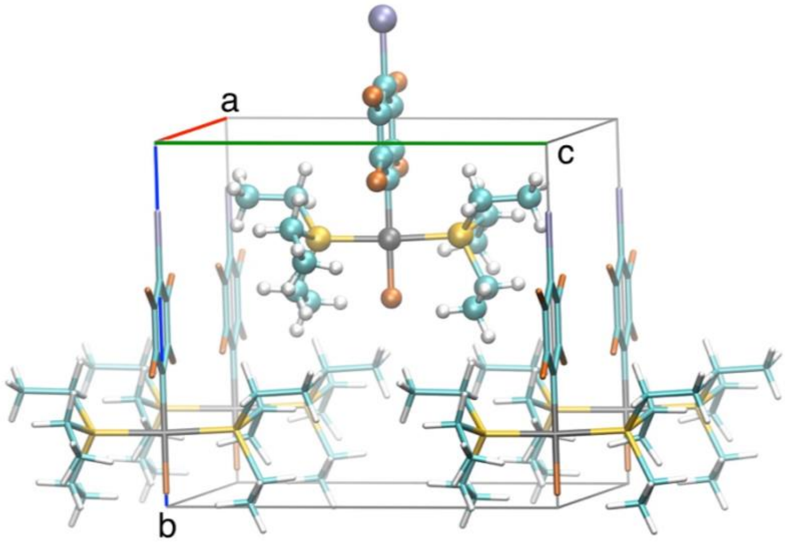

	Exptl (110 K)[a]	Calculations with PBC[b]			
		PBE0	PBE0-D3	HSE06	HSE06-D3
a [Å]	7.8675(5)	8.3209	7.4824	8.3007	7.4923
b [Å]	11.3291(3)	11.3587	11.3031	11.3506	11.3066
c [Å]	12.9347(6)	13.2153	12.7045	13.1958	12.7146
α (°)	90.000	90.000	90.000	90.000	90.000
β (°)	94.392(5)	99.175	95.2142	99.136	95.311
γ (°)	90.000	90.000	90.000	90.000	90.000
RMSD[c]	---	*0.213*	*0.180*	*0.207*	*0.177*

[a] Values as reported in reference 39. [b] Calculated using SDD(ECP) with the associated GTO basis set (Ni, I, P); pob_TZVP_rev2 basis set (F, C, H). [c] Root-mean-square deviations (RMSD) between the calculated and experimental (*a*, *b*, *c*) parameters shown in the Table.

Table S2. Experimental and calculated unit-cell parameters for **1oF** (monoclinic cell, $P2_1$ space group) with periodic boundary conditions (PBC) as a function of the functional.

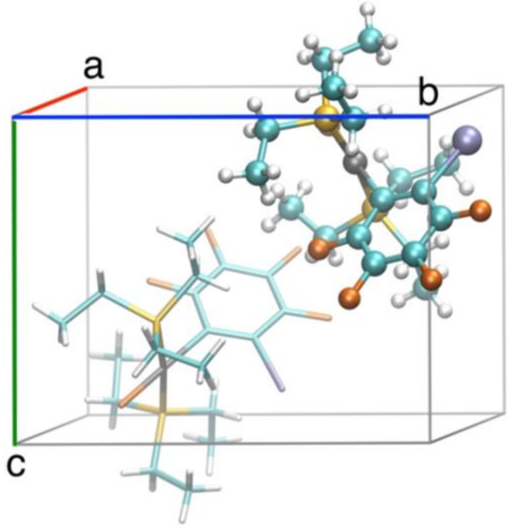

	Exptl (110 K)[a]	Calculations with PBC[b]			
		PBE0	PBE0-D3	HSE06	HSE06-D3
a [Å]	9.4116(5)	9.1975	9.0622	9.1930	9.1398
b [Å]	12.7010(8)	12.9255	12.3871	12.9066	12.3365
c [Å]	9.7270(5)	10.3809	9.5496	10.3243	9.4899
α (°)	90.000	90.000	90.000	90.000	90.000
β (°)	95.377(5)	94.267	95.235	94.031	94.948
γ (°)	90.000	90.000	90.000	90.000	90.000
RMSD[c]	---	*0.434*	*0.091*	*0.408*	*0.066*

[a] Values as reported in reference 39. [b] Calculated using SDD(ECP) with the associated GTO basis set (Ni, I, P); pob_TZVP_rev2 basis set (F, C, H). [c] Root-mean-square deviations (RMSD) between the calculated and experimental (*a*, *b*, *c*) parameters shown in the Table.

Table S3. Experimental and calculated unit-cell parameters for **3F** (orthorhombic cell, $P2_12_12_1$ space group) with periodic boundary conditions (PBC) as a function of the functional.

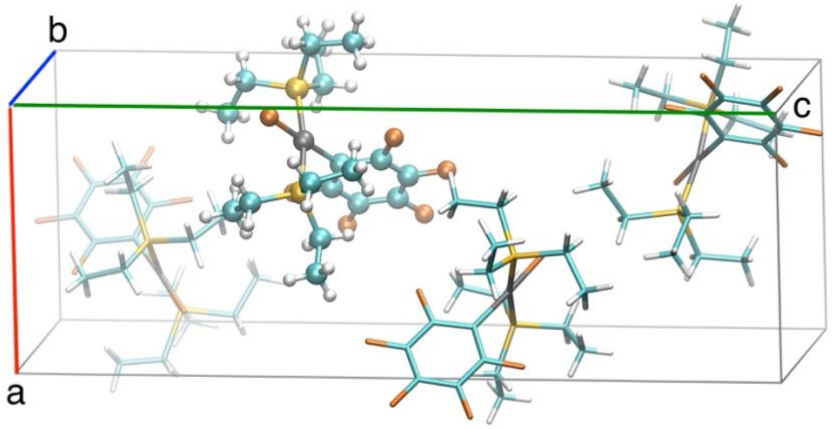

	Exptl (110 K)[a]	Calculations with PBC[b]			
		PBE0	PBE0-D3	HSE06	HSE06-D3
a [Å]	9.406(6)	9.365	8.813	9.345	8.816
b [Å]	9.447(5)	9.981	9.353	9.960	9.412
c [Å]	26.620(2)	26.317	25.855	26.281	25.832
α (°)	90.0	90.0	90.0	90.0	90.0
β (°)	90.0	90.0	90.0	90.0	90.0
γ (°)	90.0	90.0	90.0	90.0	90.0
RMSD[c]	---	*0.428*	*0.349*	*0.434*	*0.390*

[a] Values as reported in reference 42. [b] Calculated using SDD(ECP) with the associated GTO basis set (Ni, I, P); pob_TZVP_rev2 basis set (F, C, H). [c] Root-mean-square deviations (RMSD) between the calculated and experimental (*a*, *b*, *c*) parameters shown in the Table.

Table S4. Experimental and optimized structure, selected bond distances (in Å) and angles (in degrees), for **1pF** with periodic boundary conditions (PBC) as a function of the functional.

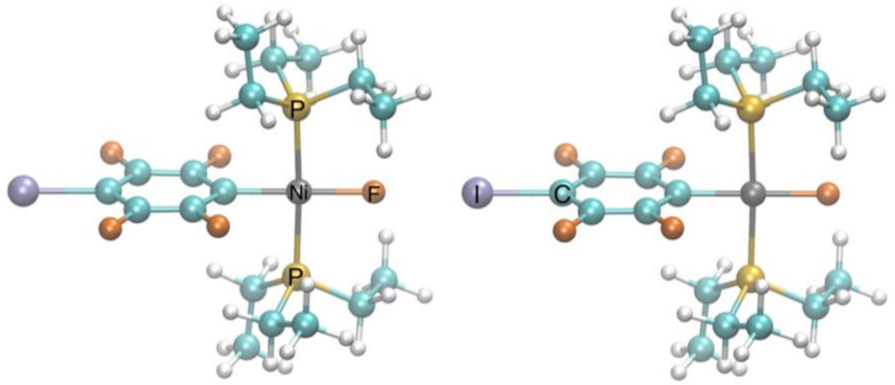

	Exptl (110 K)[a]	Optimized structure with PBC[b]			
		PBE0	PBE0-D3	HSE06	HSE06-D3
Ni–P	2.208(2)	2.2322	2.2352	2.2317	2.2363
Ni–F	1.837(5)	1.8478	1.8641	1.8497	1.8660
Ni–C	1.873(13)	1.8915	1.8950	1.8890	1.8951
F···I	2.655(5)	2.6566	2.5821	2.6477	2.5815
C–I	2.096(11)	2.1053	2.1060	2.1062	2.1069
C–I···F	180.0	180.0	180.0	180.0	180.0
Ni– F···I	180.0	180.0	180.0	180.0	180.0
F–Ni–C	180.0	180.0	180.0	180.0	180.0
P–Ni–P	177.1(1)	178.9	174.8	178.8	174.9
P–Ni–F	91.46(6)	90.56	92.60	90.60	92.55
RMSD[c]	---	*0.009*	*0.043*	*0.011*	*0.044*

[a] Values as reported in reference 39. [b] Calculated using SDD(ECP) with the associated GTO basis set (Ni, I, P); pob_TZVP_rev2 basis set (F, C, H). [c] Root-mean-square deviations (RMSD) between the calculated and experimental bond distances shown in the Table.

Table S5. Experimental and optimized structure, selected bond distances (in Å) and angles (in degrees), for **1oF** with periodic boundary conditions (PBC) as a function of the functional.

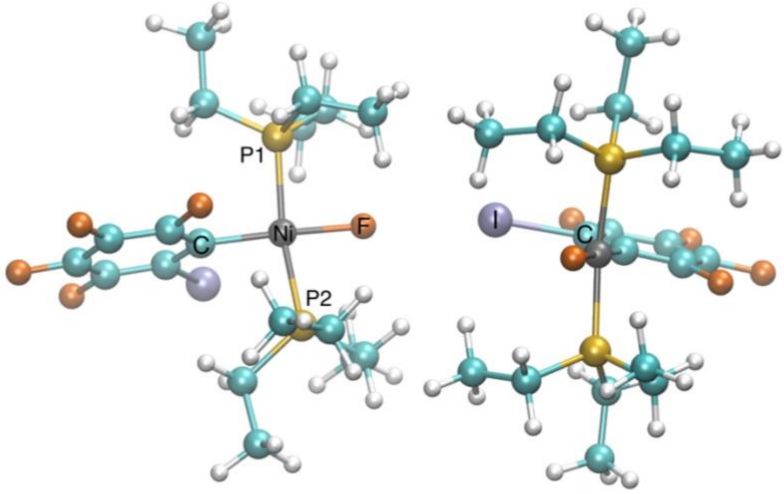

	Exptl (110 K)[a]	Optimized structure with PBC[b]			
		PBE0	PBE0-D3	HSE06	HSE06-D3
Ni–P1	2.218(2)	2.2296	2.2068	2.2296	2.2084
Ni–P2	2.202(2)	2.2356	2.2258	2.2349	2.2308
Ni–F	1.841(5)	1.8578	1.8593	1.8610	1.8612
Ni–C	1.891(7)	1.8882	1.8769	1.8868	1.8747
F···I	2.941(5)	2.9313	2.7951	2.9252	2.7894
C–I	2.110(7)	2.1193	2.1205	2.1202	2.1228
C–I···F	173.2(2)	168.0	171.6	169.0	173.0
Ni– F···I	172.4(3)	168.7	170.8	168.6	169.5
F–Ni–C	179.3(3)	179.1	179.8	178.9	179.0
P1–Ni–P2	173.12(9)	173.33	174.7	173.9	173.3
P1–Ni–F	88.6(2)	88.32	89.8	88.7	89.6
RMSD[c]	---	*0.015*	*0.064*	*0.017*	*0.067*

[a] Values as reported in reference 39. [b] Calculated using SDD(ECP) with the associated GTO basis set (Ni, I, P); pob_TZVP_rev2 basis set (F, C, H). [c] Root-mean-square deviations (RMSD) between the calculated and experimental bond distances shown in the Table.

Table S6. Experimental and optimized structure, selected bond distances (in Å) and angles (in degrees), for **3F** with periodic boundary conditions (PBC) as a function of the functional. The labelling of atoms is similar to that for **1oF**.

	Exptl (110 K)[a]	Optimized structure with PBC[b]			
		PBE0	PBE0-D3	HSE06	HSE06-D3
Ni–P1	2.198(3)	2.2248	2.2155	2.2238	2.2165
Ni–P2	2.201(2)	2.2264	2.2186	2.2254	2.2191
Ni–F	1.838(6)	1.8388	1.8469	1.8401	1.8490
Ni–C	1.879(8)	1.8923	1.9004	1.8898	1.8991
F1–Ni–C	177.4(3)	179.1	178.6	179.1	178.7
P1–Ni–P2	171.4(1)	171.5	171.0	171.5	171.0
P1–Ni–F	87.6(2)	86.7	86.5	86.6	86.3
RMSD[c]	---	*0.012*	*0.005*	*0.011*	*0.004*

[a] Values as reported in reference 42. [b] Calculated using SDD(ECP) with the associated GTO basis set (Ni, I, P); pob_TZVP_rev2 basis set (F, C, H). [c] Root-mean-square deviations (RMSD) between the calculated and experimental bond distances shown in the Table.

Table S7. Calculated ^{19}F NMR shielding (σ) and chemical shift (δ) tensor components (in ppm) for **1pF-d$_{(solid)}$** and **1oF-d$_{(solid)}$** dimers.

	**1pF**	**1pF-d$_{(solid)}$**			**1oF**	**1oF-d$_{(solid)}$**		
	GIPAW[a]	NR[b]	2c[c]	4c[d]	GIPAW[a]	NR[b]	2c[c]	4c[d]
σ_{iso}	493.1	496.0	506.3	487.5	514.4	513.5	522.9	513.9
σ_{11}	278.0	276.8	293.3	293.3	249.7	249.8	268.5	260.7
σ_{22}	364.8	355.2	373.3	357.9	412.6	407.8	421.5	412.1
σ_{33}	836.4	856.2	852.4	826.2	880.9	883.1	878.8	869.0
δ_{iso}[e]	-350.2	-357.1	-356.7	-343.2	-371.5	-374.6	-373.3	-369.7
δ_{11}[e]	-135.2	-137.8	-143.6	-149.0	-106.8	-110.8	-118.8	-116.4
δ_{22}[e]	-221.9	-216.2	-223.7	-213.6	-269.8	-268.8	-271.9	-267.8
δ_{33}[e]	-693.6	-717.2	-702.7	-682.0	-738.1	-744.1	-729.2	-724.7
RMSD[f]	*40.9*	*53.0*	*43.1*	*36.8*	*46.7*	*48.9*	*39.4*	*38.8*

[a] Calculated using the GIPAW method. [b] Obtained with the PBE functional combining the aug-pcSseg2 basis set for F and pcseg-2 basis set for the rest of the atoms, including ECP for Ni. [c] Obtained using the 2c-ZORA-PBE/TZ2P method. [d] Obtained using the 4c-DKS/PBE method and combining the Dyall's VTZ basis set for Ni, F, and I, and the uncontracted upcS-2 basis set for the rest of the atoms. [e] Values reported relative to $CFCl_3$, σ = 139.0 (1c), 149.6 (2c), and 144.3 ppm (4c). [f] Total root-mean-square deviations between the calculated and experimental shift values.

Table S8. Calculated ^{19}F NMR shielding (σ) and chemical shift (δ) tensor components (in ppm) of **1pF-m(solid)**, **1oF-m(solid)**, and **3F-m(solid)**.

	**1pF-m(solid)**			**1oF-m(solid)**			**3F-m(solid)**		
	NR[a]	2c[b]	4c[c]	NR[a]	2c[b]	4c[c]	NR[a]	2c[b]	4c[c]
σ_{iso}	523.8	537.6	529.0	557.1	544.8	562.2	530.9	547.8	536.5
σ_{11}	220.9	251.3	233.5	251.6	247.0	269.1	280.0	301.7	297.1
σ_{22}	395.0	395.2	395.3	462.2	438.4	462.0	369.7	384.0	364.5
σ_{33}	955.4	966.3	958.2	957.3	949.0	955.6	942.9	957.6	948.0
δ_{iso}	-384.8	-387.9	-384.7	-418.1	-395.2	-418.0	-391.9	-398.1	-392.3
δ_{11}	-81.9	-101.6	-89.2	-112.7	-97.4	-124.9	-141.1	-152.1	-152.9
δ_{22}	-256.0	-245.5	-251.0	-323.2	-288.8	-317.7	-230.7	-234.3	-220.2
δ_{33}	-816.5	-816.7	-814.0	-818.3	-799.4	-811.3	-803.9	-807.9	-803.7

[a] Obtained with the PBE functional combining the aug-pcSseg2 basis set for F and pcseg-2 basis set for the rest of the atoms, including ECP for Ni. [b] Obtained using the 2c-ZORA-PBE/TZ2P method. [c] Obtained using the 4c-DKS/PBE method and combining the Dyall's VTZ basis set for Ni, F, and I, and the uncontracted upcS-2 basis set for the rest of the atoms. [d] Values reported relative to $CFCl_3$, σ = 139.0 ppm (NR), 149.6 ppm (2c), 144.3 ppm (4c).

Table S9. Selected bond distances (in Å) and angles (in degrees) for **1pF-m(solv)**, **1oF-m(solv)**, and **3F-m(solv)** optimized in benzene solution using the SMD method.

	Optimized structures in benzene solution[a]		
	**1pF-m(solv)**	**1oF-m(solv)**	**3F-m(solv)**
Ni–P1	2.2341	2.2380	2.2276
Ni–P2	2.2341	2.2329	2.2275
Ni–F	1.8279	1.8337	1.8360
Ni–C	1.8901	1.8952	1.8930
C–I	2.0809	2.1039	---
F–Ni–C	180.0	178.5	180.0
P1–Ni–P2	176.6	171.7	170.4
P1–Ni–F	88.3	84.5	85.2

[a] Geometries optimized using the PBE0 functional combining the aug-pcSseg2 basis set for F and pcseg-2 basis set for the rest of the atoms, including ECP for Ni. Calculations include the SMD model for solvation in C_6H_6.

Table S10. Calculated ^{19}F NMR shielding (σ) and chemical shift (δ) tensor components (in ppm) for **1pF-m(solv)**, **1oF-m(solv)**, and **3F-m(solv)** in benzene solution. The structure of these species has been optimized in benzene solution (SMD method). The experimental isotropic chemical shifts in benzene solution are: **1pF-m(solv)** -388.3, **1oF-m(solv)** -397.9, **3F(solv)** -394.3 (ppm).

	**1pF-m(solv)**		**1oF-m(solv)**		**3F-m(solv)**	
	NR[a]	2c[b]	NR[a]	2c[b]	NR[a]	2c[b]
σ_{iso}	529.5	541.9	560.1	574.4	535.5	550.0
σ_{11}	233.5	250.7	262.3	282.5	289.3	308.0
σ_{22}	414.5	431.2	477.0	494.2	389.9	406.5
σ_{33}	940.4	443.9	941.0	946.5	927.4	935.5
δ_{iso}	-390.5	-388.4	-421.1	-420.8	-396.5	-396.4
δ_{11}	-94.5	-97.1	-123.4	-128.9	-150.3	-154.5
δ_{22}	-275.5	-292.2	-338.0	-340.6	-251.0	-253.0
δ_{33}	-801.4	-804.9	-802.0	-792.9	-788.4	-781.9

[a] Obtained with the PBE functional combining the aug-pcSseg2 basis set for F and pcseg-2 basis set for the rest of the atoms, including ECP for Ni. Calculations include the SMD model for solvation in C_6H_6 [b] Obtained at the 2c-ZORA-PBE/TZ2P level using the COSMO model for solvation in C_6H_6. [c] Values reported relative to $CFCl_3$ molecule, σ = 139.0 ppm (NR), 149.6 ppm (2c).

Table S11. Calculated shielding tensor principal components of fluorine with diamagnetic and paramagnetic plus spin-orbit contributions (in ppm) for the selected Ni–F complexes and HF···I–CH_3 model. Values calculated at the 2c-ZORA-PBE/TZ2P level.

	σ_{iso}	σ_{11}	σ_{22}	σ_{33}	σ_{11}^{dia}	σ_{22}^{dia}	σ_{33}^{dia}	σ_{11}^{p+SO}	σ_{22}^{p+SO}	σ_{33}^{p+SO}
**1pF-m(solid)**	537.6	251.3	395.2	966.3	482.8	455.3	446.6	-231.4	-60.2	519.7
**1pF-d(solid)**	506.3	293.3	373.3	852.4	494.8	455.9	447.7	-201.4	-82.6	404.5
**1oF-m(solid)**	544.8	247.0	438.4	949.0	481.2	455.3	446.7	-234.1	-16.9	502.2
**1oF-d(solid)**	522.9	268.5	421.5	878.8	489.2	453.9	444.9	-220.7	-32.4	433.9
**HF**	414.5	380.0	380.0	483.3	464.4	464.4	481.0	-84.4	-84.4	2.3
**HF···ICH₃**	394.6	345.7	345.7	492.6	461.6	461.6	489.7	-116.0	-116.0	2.9
**NiH(F)(PH₃)₂**	489.6	195.0	311.0	962.9	491.3	457.1	452.2	-296.3	-146.1	510.6

Table S12. ALMO-EDA analysis (in kJ/mol) for **1pF-d$_{(solid)}$** and **1oF-d$_{(solid)}$** considering the dimers in terms of the two monomeric units, **1pF-m$_{(solid)}$** and **1oF-m$_{(solid)}$,** computed in gas-phase at the PBE/TZ2P level. The geometries are kept constant and thus there is no structural reorganization term.

	**1pF-d$_{(solid)}$**	**1oF-d$_{(solid)}$**
Total binding (ΔE_{bind})[a]	-17.72	-8.71
Solvation (ΔE_{solv})	---	---
Frozen (ΔE_{frz})[b]	12.64	11.33
Polarization (ΔE_{pol})	-11.45	-6.24
Charge transfer (ΔE_{ct})	-18.90	-13.79
Electrostatic (ΔE_{elec})	-52.40	-32.31
Pauli (ΔE_{Pauli})	83.07	60.55
Dispersion (ΔE_{disp})	-18.03	-16.92

[a] ΔE_{bind} is the total binding energy defined as $\Delta E_{sol} + \Delta E_{frz} + \Delta E_{pol} + \Delta E_{ct}$.
[b] ΔE_{frz} is the frozen interaction defined as $\Delta E_{elec} + \Delta E_{Pauli} + \Delta E_{disp}$.

Natural Chemical Shift (NCS) description of the ^{19}F shielding tensor

Table S13. Shielding tensor components (σ^{p+SO}) in **1pF-m$_{(solid)}$** and **1pF-d$_{(solid)}$** using VB representation of the systems where Ni forms covalent bonds with fluorine and one of the phosphine ligands.

Shielding tensor component σ_{11}			
	**Monomer 1pF-m$_{(solid)}$**	**Dimer 1pF-d$_{(solid)}$**	$\Delta\sigma^{d-m}$
F (p_y) LP	-188.1	-148.01	40.09
F (p_z) LP	-11.50	-21.04	-9.54
P LP	-15.30	-15.37	-0.07
BD F – Ni	5.56	5.46	-0.10
BD Ni – P	-8.54	-8.54	0.00
sum:	**-231.4**	**-201.4**	**30.0**

Shielding tensor component σ_{22}			
	**Monomer 1pF-m$_{(solid)}$**	**Dimer 1pF-d$_{(solid)}$**	$\Delta\sigma^{d-m}$
F (sp_x) LP	-24.20	-26.60	-2.40
F (p_y) LP	-7.61	-7.52	0.09
F (p_z) LP	118.81	103.93	-14.88
Ni LP	16.36	19.23	2.87
C LP	-27.34	-24.68	2.66
BD F – Ni	-142.11	-152.93	-10.82
BD* F – Ni	11.99	12.50	0.51
BD* Ni – P	-4.42	-4.73	-0.31
I (s) LP	---	-11.21	---
I (p_z) LP	---	11.89	---
BD I – C	---	-3.41	---
BD* I – C	---	8.13	---
F (Ryd)	---	-5.33	---
sum:	**-60.2**	**-82.6**	**-22.4**

Shielding tensor component σ_{33}			
	**Monomer 1pF-m$_{(solid)}$**	**Dimer 1pF-d$_{(solid)}$**	$\Delta\sigma^{d-m}$
F (sp_x) LP	-9.09	-20.45	-11.36
F (p_y) LP	405.89	339.84	-66.05
F (p_z) LP	-9.22	-8.68	0.54
Ni LP	44.26	43.24	-1.02
P LP	27.85	25.97	-1.88
C LP	60.86	53.97	-6.89
BD F – Ni	-13.45	-48.47	-35.02
BD I – C	--	-2.34	---
BD Ni – P	14.92	15.16	0.24
BD C – H	---	-3.03	---
BD* F – Ni	14.04	14.75	0.71
BD* Ni – P	-8.14	-8.51	-0.37
I (s) LP	---	-10.47	---
I (p_y) LP	---	13.33	---
BD* I – C	---	7.26	---
F (Ryd)	---	-4.56	---
sum:	**519.7**	**404.5**	**-115.2**

Table S14. Shielding tensor components in **1oF-m(solid)** and **1oF-d(solid)**. The valence representation is similar to that for **1pF**.

Shielding tensor component along σ_{11}			
	**Monomer 1oF-m(solid)**	**Dimer 1oF-d(solid)**	$\Delta\sigma^{\mathrm{d-m}}$
F (sp_x) LP	---	-69.57	---
F (p_y) LP	-148.81	-44.54	104.27
F (p_z) LP	-54.09	-76.69	-22.60
P LP	-12.61	-13.85	-1.24
BD F – Ni	5.61	5.29	-0.32
BD Ni – P	-8.07	-7.37	0.70
sum:	**-234.1**	**-220.7**	**13.4**

Shielding tensor component along σ_{22}			
	**Monomer 1oF-m(solid)**	**Dimer 1oF-d(solid)**	$\Delta\sigma^{\mathrm{d-m}}$
F (sp_x) LP	-23.46	-10.53	12.93
F (p_y) LP	28.94	61.80	32.86
F (p_z) LP	87.49	38.16	-49.33
Ni LP	16.58	17.42	0.84
C LP	-20.40	-18.97	1.43
BD F – Ni	-119.02	-134.46	-15.44
BD* F – Ni	12.74	12.85	0.11
BD* Ni – P	-4.61	-4.77	-0.16
I (s) LP	---	-5.00	---
I (p_z) LP	---	6.29	---
BD* I – C	---	5.08	---
sum:	**-16.9**	**-32.4**	**-15.5**

Shielding tensor component σ_{33}			
	**Monomer 1oF-m(solid)**	**Dimer 1oF-d(solid)**	$\Delta\sigma^{\mathrm{d-m}}$
F (sp_x) LP	-8.96	144.92	153.88
F (p_y) LP	291.63	50.00	-241.63
F (p_z) LP	94.34	139.43	45.09
Ni LP	44.80	43.47	-1.33
P LP	24.64	21.98	-2.66
C LP	44.43	41.54	-2.89
BD F – Ni	---	-21.58	---
BD Ni – P	13.76	13.71	-0.05
BD* F – Ni	13.41	13.32	-0.09
BD* Ni – P	-7.35	-7.48	-0.13
I (s) LP	---	-5.66	---
I (p_y) LP	---	6.24	---
sum:	**502.2**	**433.9**	**-68.30**

Table S15. NBO analysis of selected canonical molecular orbitals for **1pF-m**$_{\mathbf{(solid)}}$. The labeling of atoms is that from the calculations. Ni is Ni2, F is F5, etc.

LUMO +5; E= -0.0242	LUMO +4; E= -0.0363
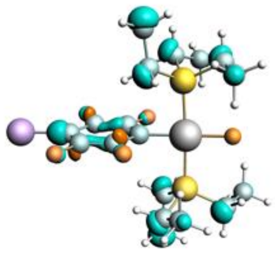	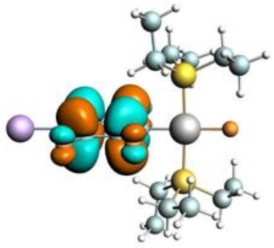
-0.218*[830]: RY (1) H28(ry)	0.640*[165]: BD*(2) C11- C25*<br>0.334*[105]: BD (2) C10- C12<br>0.331*[205]: BD*(2) C26- C27*<br>0.312*[163]: BD*(2) C10- C12*<br>-0.300*[147]: BD (2) C26- C27
LUMO +3; E= -0.0390	LUMO +2; E= -0.0530
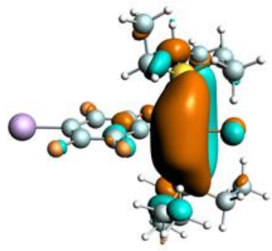	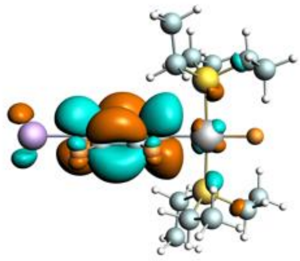
0.326*[152]: BD*(1) P3- C17*<br>0.325*[155]: BD*(1) P4- C18*<br>-0.242*[154]: BD*(1) P4- C14*<br>-0.240*[151]: BD*(1) P3- C13*<br>-0.225*[265]: RY (3) Ni2(ry)<br>-0.224*[452]: RY (1) C10(ry)	0.552*[205]: BD*(2) C26- C27*<br>-0.516*[163]: BD*(2) C10- C12*<br>0.366*[107]: BD (2) C11- C25
LUMO +1; E= -0.0754	LUMO; E= -0.0888
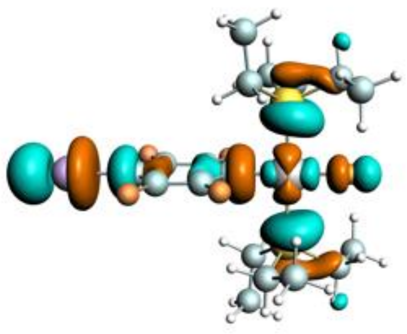	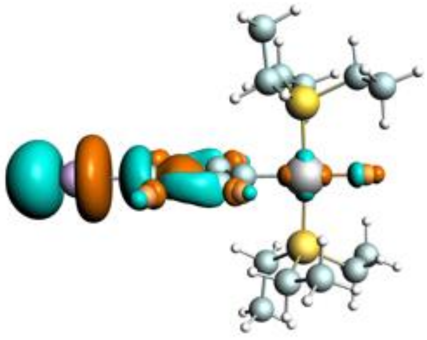
0.575*[148]: BD*(1) I1- C27*<br>0.410*[150]: BD*(1) Ni2- F5*<br>-0.406*[149]: BD*(1) Ni2- P3*<br>-0.315*[89]: LP (1) C10(lp)<br>0.277*[73]: LP (1) P4(lp)	-0.652*[148]: BD*(1) I1- C27*<br>0.332*[150]: BD*(1) Ni2- F5*<br>-0.320*[149]: BD*(1) Ni2- P3*

HOMO; E= -0.1644	HOMO-1; E= -0.1645
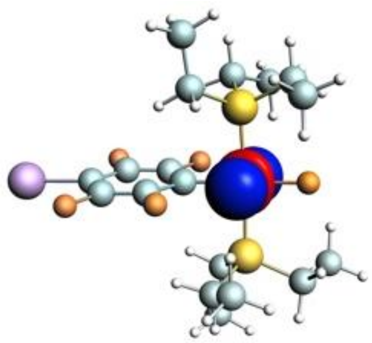	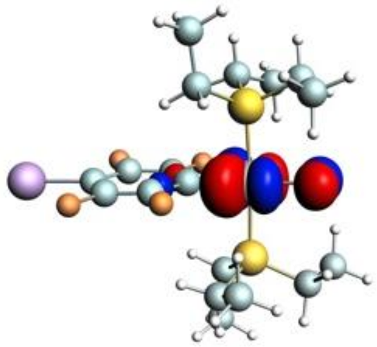
0.967*[69]: LP (1) Ni2(lp)	0.929*[70]: LP (2) Ni2(lp)<br>0.337*[76]: LP (3) F5(lp)
HOMO-2; E= -0.1845	HOMO-3; E= -0.1859
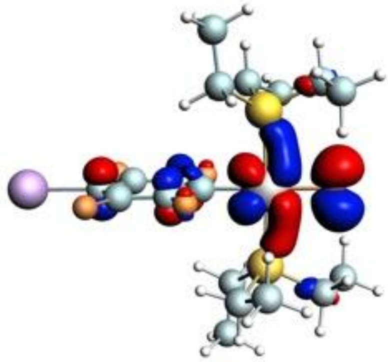	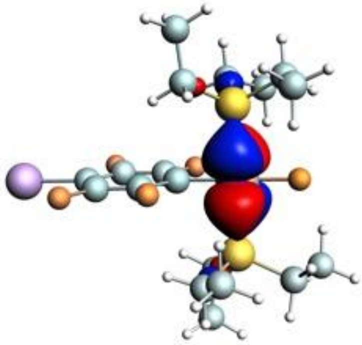
0.786*[72]: LP (4) Ni2(lp)<br>0.455*[75]: LP (2) F5(lp)	0.973*[71]: LP (3) Ni2(lp)
HOMO-4; E= -0.2084	HOMO-5; E= -0.2172
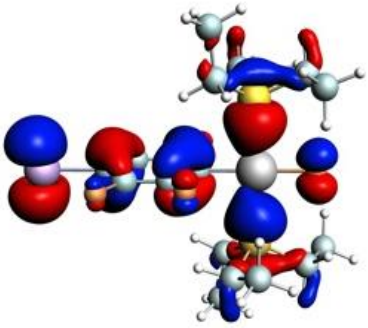	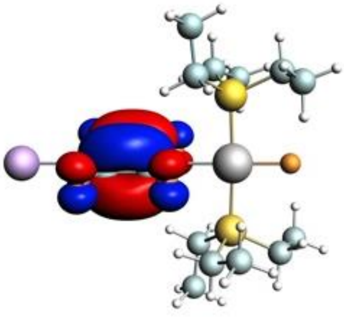
0.412*[68]: LP (3) I1(lp)<br>0.360*[73]: LP (1) P4(lp)<br>-0.341*[105]: BD (2) C10- C12<br>-0.329*[91]: BD (1) Ni2- P3<br>0.323*[147]: BD (2) C26- C27<br>0.292*[75]: LP (2) F5(lp)<br>0.234*[165]: BD*(2) C11- C25*	0.602*[107]: BD (2) C11- C25<br>-0.319*[205]: BD*(2) C26- C27*<br>0.307*[105]: BD (2) C10- C12<br>0.290*[147]: BD (2) C26- C27<br>0.287*[163]: BD*(2) C10- C12*<br>0.261*[88]: LP (3) F9(lp)<br>0.261*[85]: LP (3) F8(lp)<br>0.254*[82]: LP (3) F7(lp)<br>0.254*[79]: LP (3) F6(lp)

Table S16. NBO analysis of selected canonical molecular orbitals for **1pF-d(solid)**. The labeling of atoms is that from the calculations. The left-side molecule has labels, F9, Ni3, I2, and the right-side molecule has labels F10, Ni4.

LUMO +1; E= -0.0834	LUMO; E= -0.0966
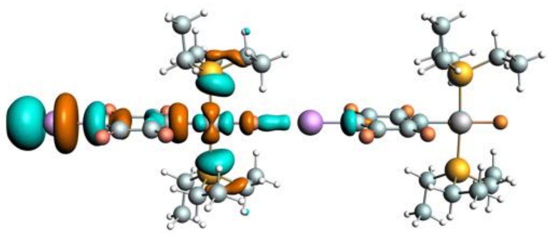	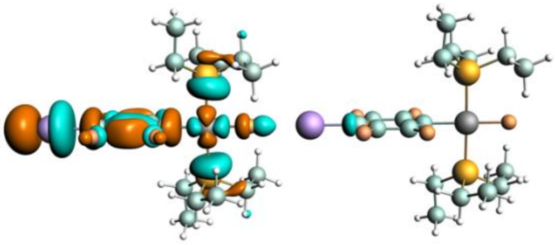
-0.708*[296]: BD*(1) I2- C31*<br>-0.293*[298]: BD*(1) Ni 3- F9*<br>0.274*[297]: BD*(1) Ni 3- P5*<br>0.249*[178]: LP (1) C20(lp)	-0.488*[296]: BD*(1) I 2- C31*<br>0.415*[298]: BD*(1) Ni3- F9*<br>-0.391*[297]: BD*(1) Ni3- P5*<br>-0.271*[178]: LP (1) C20(lp)<br>0.265*[145]: LP (1) P6(lp)
HOMO; E= -0.1596	HOMO-1; E= -0.1597
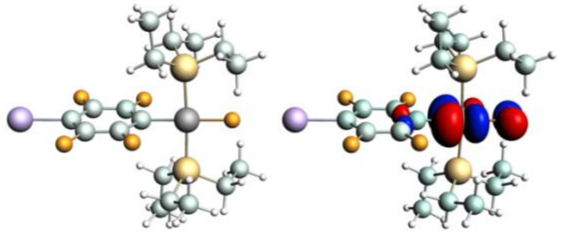	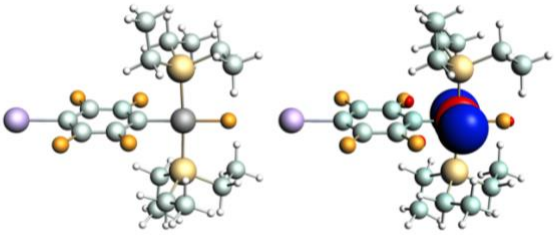
-0.930*[142]: LP (2) Ni4(lp)<br>-0.334*[152]: LP (3) F10(lp)	-0.967*[141]: LP (1) Ni4(lp)
HOMO-2; E= -0.1729	HOMO-3; E= -0.1745
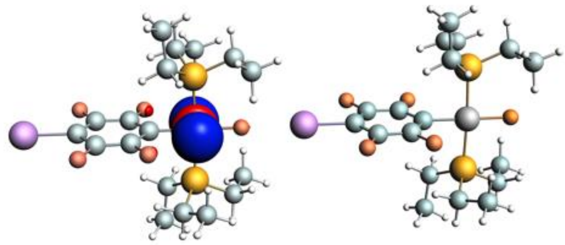	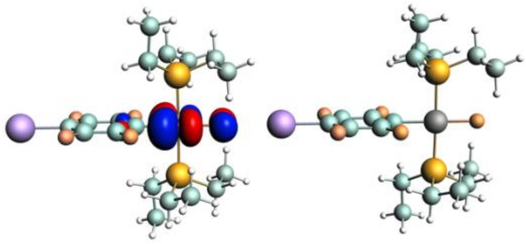
-0.968*[137]: LP (1) Ni3(lp)	0.939*[138]: LP (2) Ni3(lp)<br>0.301*[148]: LP (2) F9(lp)
HOMO-4; E= -0.1787	HOMO-5; E= -0.1812
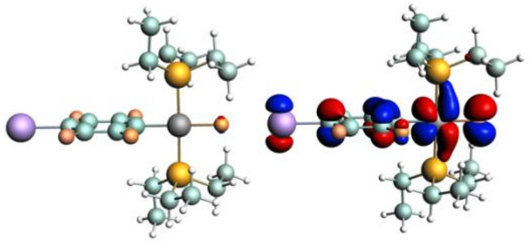	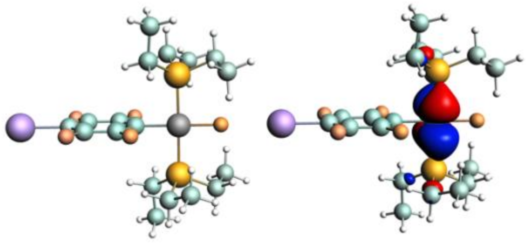
-0.773*[144]: LP (4) Ni4(lp)<br>-0.419*[151]: LP (2) F10(lp)<br>-0.230*[207]: BD (2) C19- C23	-0.973*[143]: LP (3) Ni4(lp)

HOMO-6; E= -0.1941	HOMO-7; E= -0.1945
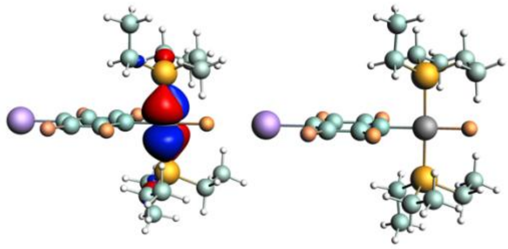	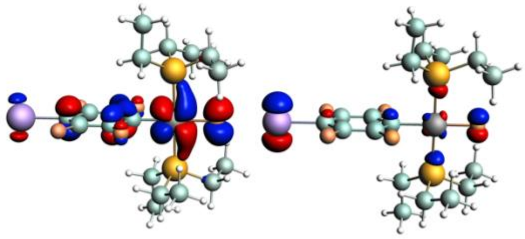
-0.972*[139]: LP (3) Ni3(lp)	0.727*[140]: LP (4) Ni3(lp)<br>0.361*[147]: LP (1) F9(lp)<br>-0.234*[133]: LP (3) I1(lp)
HOMO-8; E= -0.2006	HOMO-9; E= -0.2081
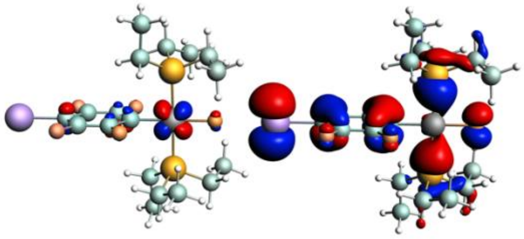	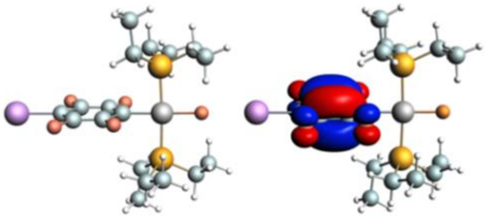
0.422*[133]: LP (3) I1(lp)<br>0.319*[140]: LP (4) Ni 3(lp)<br>0.307*[146]: LP (1) P 8(lp)<br>0.298*[219]: BD (2) C26- C28<br>-0.296*[207]: BD (2) C19- C23<br>0.291*[151]: LP (2) F10(lp)<br>-0.279*[183]: BD (1) Ni4- P7	-0.603*[212]: BD (2) C21- C25<br>0.315*[335]: BD*(2) C26- C28*<br>-0.308*[207]: BD (2) C19- C23<br>-0.291*[219]: BD (2) C26- C28<br>-0.291*[323]: BD*(2) C19- C23*<br>-0.259*[167]: LP (3) F15(lp)<br>-0.259*[170]: LP (3) F16(lp)<br>-0.253*[161]: LP (3) F13(lp)<br>-0.253*[155]: LP (3) F11(lp)
HOMO-10; E= -0.2159	HOMO-11; E= -0.2226
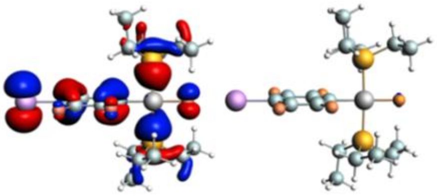	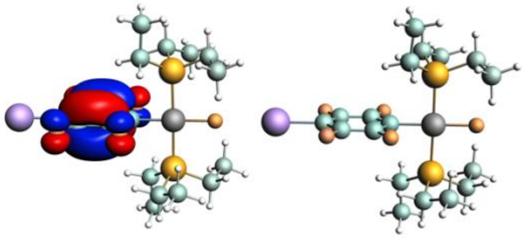
0.408*[136]: LP (3) I2(lp)<br>0.362*[145]: LP (1) P6(lp)<br>-0.331*[181]: BD (1)Ni3- P5<br>-0.306*[210]: BD (2) C20- C24<br>0.300*[222]: BD (2) C29- C31<br>0.282*[147]: LP (1) F9(lp)	0.602*[214]: BD (2) C22- C27<br>-0.318*[338]: BD*(2) C29- C31*<br>0.306*[210]: BD (2) C20- C24<br>0.289*[222]: BD (2) C29- C31<br>0.288*[326]: BD*(2) C20- C24*<br>0.261*[173]: LP (3) F17(lp)<br>0.261*[176]: LP (3) F18(lp)<br>0.255*[158]: LP (3) F12(lp)<br>0.255*[164]: LP (3) F14(lp)

Table S17. Canonical molecular orbitals of **H–F** model structure.

LUMO+1; E= 0.0767	LUMO; E= -0.0297	HOMO; E= -0.3555
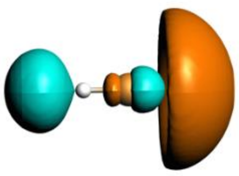	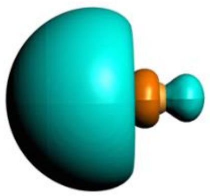	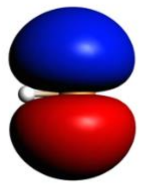
HOMO; E= -0.3555	HOMO-1; E= -0.4962	HOMO-2; E= -1.1081
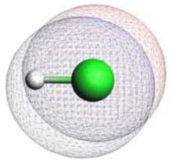	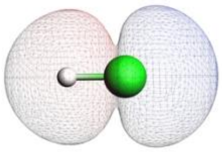	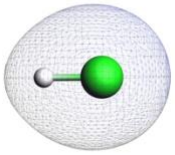

Table S18. NBO analysis of selected canonical molecular orbitals for **HF···ICH$_3$** model structure. The labeling of atoms is that from the calculations. F is F1, H(F) is H4, I is I2, etc.

LUMO+5; E= 0.0834	LUMO+4; E= 0.0560	LUMO+3; E= 0.0560
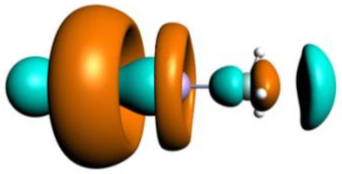	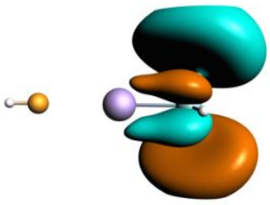	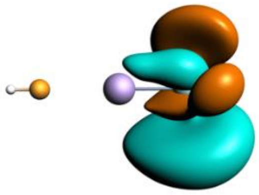
-0.458*[63]: RY (1) I 2(ry)<br>-0.306*[69]: RY (7) I 2(ry)<br>0.305*[46]: RY (5) F 1(ry)<br>-0.297*[108]: RY (46) I 2(ry)<br>-0.230*[37]: BD*(1) F1- H 4*<br>0.227*[143]: RY (3) H 4(ry)	0.540*[151]: RY (1) H 5(ry)<br>-0.376*[161]: RY (1) H 6(ry)<br>-0.358*[39]: BD*(1) C3- H 5*<br>0.250*[40]: BD*(1) C 3- H 6*<br>0.245*[125]: RY (6) C 3(ry)	0.529*[171]: RY (1) H 7(ry)<br>0.406*[161]: RY (1) H 6(ry)<br>-0.351*[41]: BD*(1) C3- H 7*<br>0.270*[40]: BD*(1) C 3- H 6*<br>-0.245*[126]: RY (7) C 3(ry)
LUMO+2; E= 0.0165	LUMO+1; E= -0.0207	LUMO; E= -0.0549
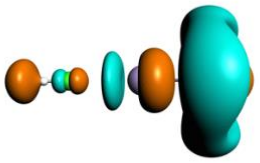	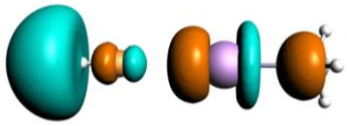	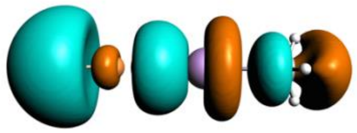

0.454*[38]: BD*(1) I 2- C 3*<br>-0.362*[171]: RY (1) H 7(ry)<br>-0.362*[161]: RY (1) H 6(ry)<br>-0.362*[151]: RY (1) H 5(ry)	-0.424*[38]: BD*(1) I 2- C 3*<br>0.340*[37]: BD*(1) F 1- H 4*<br>-0.335*[143]: RY (3) H 4(ry)<br>0.312*[46]: RY (5) F 1(ry)<br>0.294*[144]: RY (4) H 4(ry)<br>-0.236*[171]: RY (1) H 7(ry)<br>-0.236*[161]: RY (1) H 6(ry)<br>-0.236*[151]: RY (1) H 5(ry)<br>0.225*[42]: RY (1) F 1(ry)	-0.722*[38]: BD*(1) I 2- C 3*<br>-0.339*[37]: BD*(1) F1- H4*<br>-0.235*[144]: RY (4) H 4(ry)<br>-0.230*[46]: RY (5) F 1(ry)<br>0.225*[143]: RY (3) H 4(ry)
HOMO; E= -0.2134	HOMO; E= -0.2134	HOMO-1; E= -0.3070
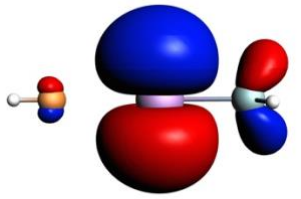	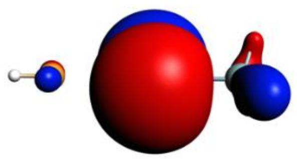	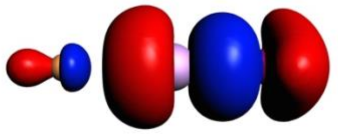
0.978*[31]: LP (3) I 2(lp)	0.978*[30]: LP (2) I 2(lp)	-0.844*[33]: BD (1) I 2- C 3<br>-0.418*[29]: LP (1) I 2(lp)
HOMO-2; E= -0.3727	HOMO-3; E= -0.3727	HOMO-4; E= -0.3806
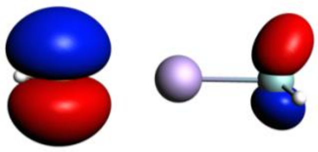	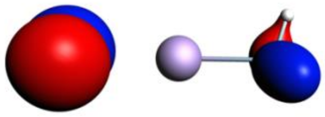	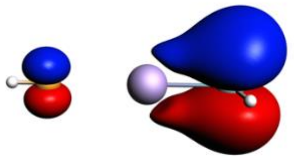
0.983*[26]: LP (1) F 1(lp)	-0.983*[27]: LP (2) F 1(lp)	0.722*[36]: BD (1) C 3- H 7<br>-0.649*[35]: BD (1) C 3- H 6
HOMO-5; E= -0.3807	HOMO-6; E= -0.5162	HOMO-7; E= -0.5783
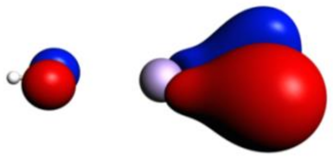	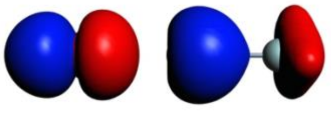	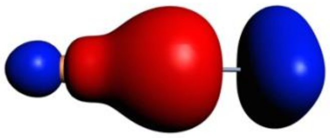
0.791*[34]: BD (1) C 3- H 5<br>-0.459*[35]: BD (1) C 3- H 6<br>-0.333*[36]: BD (1) C 3- H 7	-0.717*[32]: BD (1) F 1- H 4<br>0.599*[28]: LP (3) F 1(lp)	-0.718*[29]: LP (1) I 2(lp)<br>-0.354*[34]: BD (1) C 3- H 5<br>-0.354*[36]: BD (1) C 3- H 7<br>-0.354*[35]: BD (1) C 3- H 6<br>-0.257*[32]: BD (1) F 1- H 4
HOMO-8; E= -0.6733	HOMO-9; E= -1.1248	HOMO-10; E=-1.7970
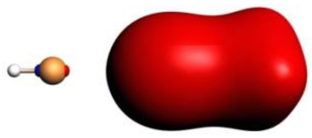	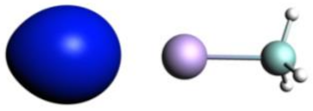	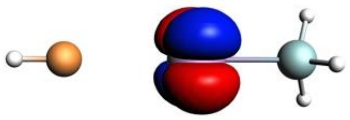
0.510*[29]: LP (1) I 2(lp)<br>-0.488*[33]: BD (1) I 2- C 3<br>-0.407*[34]: BD (1) C 3- H 5<br>-0.407*[35]: BD (1) C 3- H 6<br>-0.407*[36]: BD (1) C 3- H 7	0.766*[28]: LP (3) F 1(lp)<br>0.639*[32]: BD (1) F 1- H 4	-1.000*[16]: CR (15) I 2(cr)

Relationship between chemical shift and frontier molecular orbitals

The shielding is directly related to the electronic structure of a nucleus and in a nonrelativistic formalism can be split into diamagnetic (σ^{dia}) and paramagnetic contributions (σ^{p}) (eq 1):

$$\sigma = \sigma^{dia} + \sigma^{p} \qquad (1)$$

The diamagnetic contribution depends on the ground-state electron density and the paramagnetic term is associated with magnetic interactions between occupied and unoccupied orbitals.[1] The paramagnetic contribution to each principal component σ_{ii} (i = 1, 2, 3) can be interpreted based on Ramsey's formalism (eq 2):[2-3]

$$\sigma_{ii}^{p} \Leftrightarrow \frac{\langle\Psi_{occ}|\hat{L}_i|\Psi_{vac}\rangle\langle\Psi_{vac}|\hat{L}_i/r^3|\Psi_{occ}\rangle}{\Delta E_{vac-occ}} \qquad (2)$$

This equation describes the magnetic coupling due to the interaction of occupied (Ψ_{occ}) and vacant (Ψ_{vac}) orbitals associated with the energy E_{occ} and E_{vac}, respectively. The angular momentum operator ($\hat{L}_i$) in the first matrix element represents the interaction of the external field with electron i (orbital Zeeman term); the operator ($\hat{L}_i/r^3$) in the second term represents the interaction of electron i with the nuclear spin (paramagnetic spin-orbit term).

According to eq 2, deshielding in the direction σ_{ii} depends on the orbitals, which can be coupled by the corresponding $\hat{L}_i$ operator. The contributions of individual orbitals to deshielding can be obtained from quantum chemical calculations. Interpretation can be carried out based on canonical (molecular orbital, MO) or localized orbitals as, in particular, obtained by a natural chemical shift (NCS) analysis. Since deshielding originates from the coupling of occupied and vacant orbitals, it is observed if the p-component of the occupied and vacant MOs are perpendicular to each other and to $\hat{L}_i$ (the direction of σ_{ii}).[4]

[1] Kaupp, M.. Interpretation of NMR Chemical Shifts. In *Calculation of NMR and EPR Parameters*; Kaupp, M., Bühl, M., Malkin, V. G., Eds.; Wiley-VCH: Weinheim, 2004; pp 293-306.
[2] Ramsey, N. F.; Magnetic Shielding of Nuclei in Molecules. *Physical Review* **1950,** 78, 699-703.
[3] Pople, J. A.; The theory of carbon chemical shifts in N.M.R. *Mol. Phys.* **1964,** 7, 301-306.
[4] Gordon, C. P.; Raynaud, C.; Andersen, R. A.; Copéret, C.; Eisenstein, O., Carbon-13 NMR Chemical Shift: A Descriptor for Electronic Structure and Reactivity of Organometallic Compounds. *Acc. Chem. Res.* **2019,** *52*, 2278-2289.

Orbital Rotation Model approach

The orbital rotation model describes how occupied and empty orbitals are coupled via the appropriate angular momentum operator to induce a paramagnetic term in the direction of the angular momentum. An essential point is that both the occupied and empty orbitals are perpendicular to the direction of the angular momentum operator. These couplings appear as the main contributions to the σ^{p+SO} term since the SO term (still included) is relatively small, at least in the studied systems. The magnitude of the paramagnetic term is inversely proportional to the energy gap between the orbitals and proportional to the "overlap" between the rotated occupied orbital and the empty orbital. If both occupied and empty orbitals have an important contribution on the nuclear active atom, the paramagnetic contribution is usually negative (*i.e.,* deshielding).

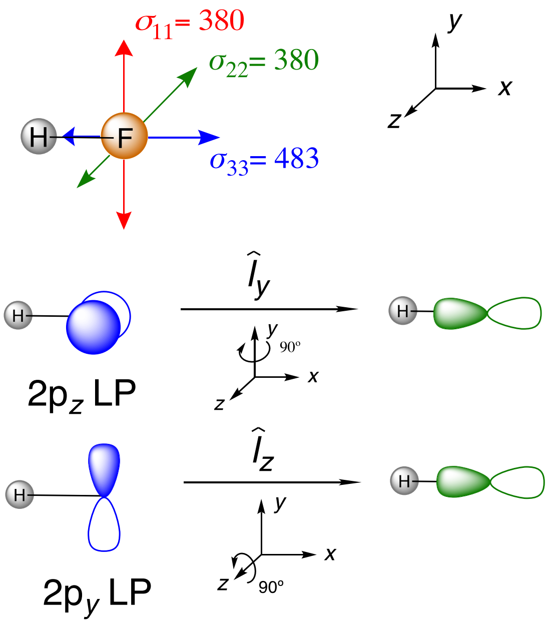

Figure S1. Schematic description of paramagnetic shielding (σ, ppm) for the case of H–F molecule. A coupling between a 2p$_z$ (or 2p$_y$) lone pair and $\sigma^*_{\mathrm{H-F}}$ results in deshielding along direction y (or z). An unconventional set of axes with x along H–F is used for easier comparison with the nickel-fluoride complexes.

As shown in Figure S1, the action of magnetic field along the *y* direction can be described by the rotation of the occupied F $2p_z$ lone pair (LP) around the *y* axis by 90°. Thus, the rotated occupied F $2p_z$ LP, now aligned with the x axis, overlaps with the empty $\sigma^*_{\mathrm{H-F}}$, inducing a paramagnetic term in the y direction. Similar reasoning applies to the F $2p_y$ LP and a rotation around the *z* axis, resulting in a paramagnetic term along the z direction. However, for a rotation around the *x* axis, the $2p_z$ (or $2p_y$) does not overlap with any empty orbital. This leads to greater paramagnetic terms (in this case deshielding) in directions perpendicular to H–F. This accounts well for the three principal components of the tensor in H–F (σ_{33} = 483 ppm (along *x*), σ_{11} and σ_{22} = 380 ppm along directions perpendicular to H–F). See Table S11 for full details.

Figure S2. HF···ICH_3 model. The F···I distance associated with the XB bond was set equal to that in **1pF-d** and the rest of structural parameters were optimized at the PBE0/TZ2P level.

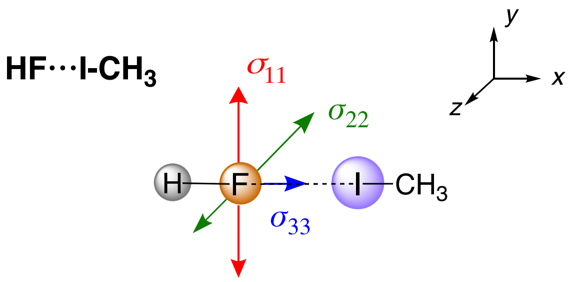

Analysis of the shielding components in Cl–F

Calculations carried out using the ORCA software[5] at the PBE/pcSseg-2[6-7] level confirm that the most important terms to the paramagnetic terms for F and Cl in Cl–F are due to the HOMO (antibonding combination of Cl and F lone pairs, with 76% on Cl and 24% on F) coupled with the LUMO σ* antibonding Cl–F co-axial orbital with 66% on Cl and 34% on F). Because both coupled orbitals have their major contribution on Cl, the paramagnetic term of the shielding at both Cl and F is dominated by the chlorine contribution with values of –2076 for Cl and +976 ppm for F. This leads to the usual deshielding at Cl, but to a shielding at F.

The analogy with the nickel-fluoride complex is the following. The $\hat{l}_z$ operator couples the HOMO [c1(d_{xyNi}) – c2(p_{yF})] with c1 > c2 with the LUMO [c'1($d_{x2-y2Ni}$) – c'2(p_{zF})] with c'1 > c'2. Importantly, the $\hat{l}_z$ operator transforms d_{xy} into d_{x2-y2} so that the magnetic coupling of these two orbitals is dominated by the Ni contribution. Thus, similarly to what was obtained for Cl–F, F is shielded by the paramagnetic contribution on Ni.

[5] Neese, F.; Wennmohs, F.; Becker, U.; Riplinger, C., The ORCA quantum chemistry program package. *J. Chem. Phys.* **2020**, *152*, 224108.

[6] Jensen, F., Segmented Contracted Basis Sets Optimized for Nuclear Magnetic Shielding. *J. Chem. Theory Comput.* **2015**, 11, 132-138.

[7] Weigend, F., Accurate Coulomb-fitting basis sets for H to Rn. *Phys. Chem. Chem. Phys.* **2006**, 8, 1057-1065.
